# Modulation of myosin by cardiac myosin binding protein-C peptides improves cardiac contractility in ex-vivo experimental heart failure models

**DOI:** 10.1038/s41598-022-08169-1

**Published:** 2022-03-14

**Authors:** Luqia Hou, Mohit Kumar, Priti Anand, Yinhong Chen, Nesrine El-Bizri, Chad J. Pickens, W. Michael Seganish, Sakthivel Sadayappan, Gayathri Swaminath

**Affiliations:** 1 Cardiometabolic Department, Merck & Co., Inc., 213 East Grand Ave., South San Francisco, CA 94080 USA; 2 Analytical R&D, Merck & Co., Inc., South San Francisco, CA 94080 USA; 3 Discovery Chemistry, Merck & Co., Inc., South San Francisco, CA 94080 USA; 4 Division of Cardiovascular Health and Disease, Department of Internal Medicine, Heart, Lung and Vascular Institute, University of Cincinnati, Cincinnati, OH 45267 USA

**Keywords:** Biochemistry, Biophysical chemistry, Immunochemistry, Structural biology

## Abstract

Cardiac myosin binding protein-C (cMyBP-C) is an important regulator of sarcomeric function. Reduced phosphorylation of cMyBP-C has been linked to compromised contractility in heart failure patients. Here, we used previously published cMyBP-C peptides 302A and 302S, surrogates of the regulatory phosphorylation site serine 302, as a tool to determine the effects of modulating the dephosphorylation state of cMyBP-C on cardiac contraction and relaxation in experimental heart failure (HF) models in vitro. Both peptides increased the contractility of papillary muscle fibers isolated from a mouse model expressing cMyBP-C phospho-ablation (cMyBP-C^AAA^) constitutively. Peptide 302A, in particular, could also improve the force redevelopment rate (*k*_tr_) in papillary muscle fibers from cMyBP-C^AAA^ (nonphosphorylated alanines) mice. Consistent with the above findings, both peptides increased ATPase rates in myofibrils isolated from rats with myocardial infarction (MI), but not from sham rats. Furthermore, in the cMyBP-C^AAA^ mouse model, both peptides improved ATPase hydrolysis rates. These changes were not observed in non-transgenic (NTG) mice or sham rats, indicating the specific effects of these peptides in regulating the dephosphorylation state of cMyBP-C under the pathological conditions of HF. Taken together, these studies demonstrate that modulation of cMyBP-C dephosphorylation state can be a therapeutic approach to improve myosin function, sarcomere contractility and relaxation after an adverse cardiac event. Therefore, targeting cMyBP-C could potentially improve overall cardiac performance as a complement to standard-care drugs in HF patients.

## Introduction

Cardiac myosin binding protein-C (cMyBP-C) is a 140-kDa sarcomeric thick filament protein which localizes in regular intervals at the C zone of the sarcomere to regulate sarcomere structure and function in the heart^1,2^. The regulation of cMyBP-C arises from three phosphorylation sites at the M-domain and the interaction of its N-terminal-region with both myosin and actin^3,4^. While cMyBP-C is highly phosphorylated under normal physiological conditions, its phosphorylation levels drop in diseased states^5^. This can be observed in both preclinical heart failure (HF) models and, clinically, in patients with hypertrophic cardiomyopathy (HCM), HF or atrial fibrillation^6^. Despite the clear benefits of targeting cMyBP-C, no drug is currently available to directly modify dephosphorylated cMyBP-C to improve cardiac function.

Human and mouse cMyBP-C proteins have three major phosphorylation sites, more than 17 sites in total^7,8^. These are located in the M-domain at the N-terminus of the molecule, and all are absent from the skeletal muscle isoform^9^. The region of the N-terminus binds to the S2 segment of myosin, close to the lever-arm domain^10,11^. This interaction can be dynamically regulated by phosphorylation/dephosphorylation of cMyBP-C. Under physiological conditions, phosphorylated cMyBP-C facilitates the activation of cross-bridge cycling, tethering the thick and thin sarcomeric filaments^12^. However, when cMyBP-C dephosphorylates in diseased conditions, it strongly interacts with myosin, thus preventing its force-generating interaction with actin^13–15^. Similarly, the catalytic cleavage of the N-terminal region of cMyBP-C during MI reduces the number of phosphorylation sites, leading to depressed cardiac contractility and function^16^.

Previous studies have determined the necessity and sufficiency of cMyBP-C phosphorylation for regulating normal cardiac function^17–20^. These studies used cardiac-specific transgenic (TG) mice expressing phospho-ablated cMyBP-C at Ser-273-Ala/Ser-282-Ala/Ser-302-Ala sites (AAA) or phospho-mimetic cMyBP-C at Ser-273-Asp/Ser-282-Asp/Ser-302-Asp (DDD) compared to non-transgenic (NTG) control mice. Therefore, to therapeutically target cMyBP-C to improve cardiac function after an adverse cardiac event, we used preclinical models, as reported in the literature. In the first model, all three serine phosphorylation sites (273, 282 and 302) were mutated to non-phosphorylatable alanines (AAA)^18^. In another model, they were mutated to phospho-mimetic aspartic acids (DDD)^19^ to mimic the physiological state of the heart. AAA replacement resulted in depressed cardiac function such that the protein could not rescue the cMyBP-C null phenotype^18^, while the DDD substitution was able to rescue the cMyBP-C null phenotype^19^. The transgenic mouse expressed cMyBP-C wild-type served as NTG controls.

Here, we tested the peptides designed based on the M-domain of cMyBP-C^21^. We hypothesized that the peptides would likely interrupt the interaction of cMyBP-C and myosin, thus improving myosin head interaction with the thin filament and, hence, power stroke. The cMyBP-C peptides tested could disrupt the tight interaction between cMyBP-C and myosin, releasing the thick filaments to resume their interaction with the thin filaments to improve muscle contractility. Indeed, permeabilizing 302A, surrogate of phosphorylation site at 302, peptide inhibited myosin from interacting with cMyBP-C, resulting in its movement toward actin, which in turn resulted in the formation of actomyosin interactions and cross-bridges, thus enhancing contractility. Taken together, the evaluation of peptide 302A in vitro led to the discovery of improved force generation and calcium sensitivity in cardiac muscle fibers. However, the mechanism(s) underlying the improvement of contractile kinetics relative to the phosphorylation status of cMyBP-C has not been fully elucidated. Under pathological conditions, we hypothesized, as suggested above, that peptide 302A modifies the binding properties of cMyBP-C in its dephosphorylated state to improve cardiac kinetics. To test this hypothesis, the effects of cMyBP-C peptides 302A and 302S were evaluated in three different in vitro assays: a steady-state ATPase assay, a myofibril ATPase assay, and a papillary muscle assay. Data generated from in vitro assays and preclinical HF models provide strong evidence that modulating cMyBP-C and, consequently, its phosphorylation status, increases ATPase activity and force kinetics in a manner that improves overall cardiac performance.

## Results

### cMyBP-C phosphorylation and total cMyBP-C were reduced in the rat MI heart failure model

**Figure 1:**
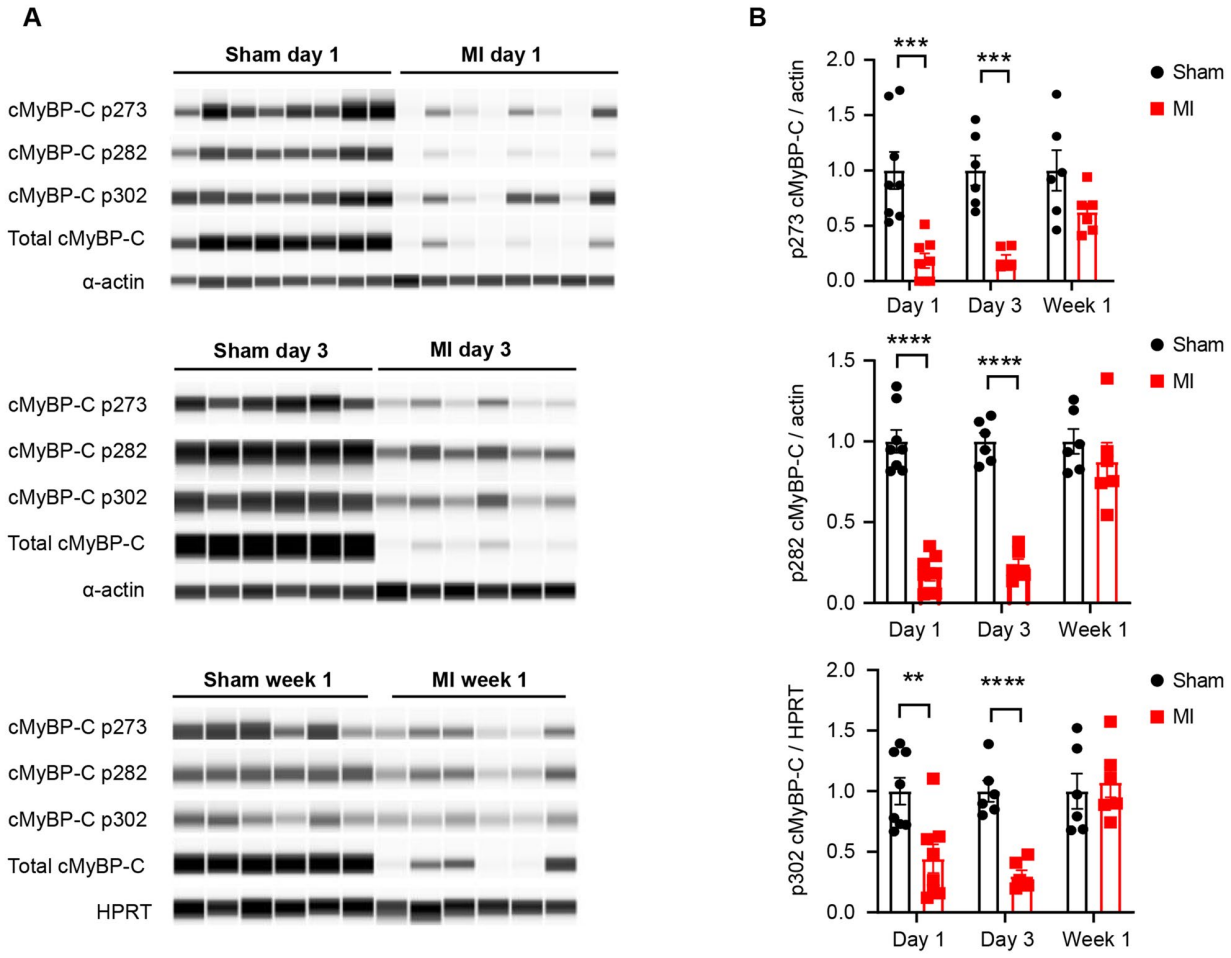
cMyBP-C phosphorylation was reduced at day 1 and day 3, but not at week 1 in post-MI rats compared to sham. (**A**) Western blot (Wes) of phosphorylated cMyBP-C tested with site-specific phospho cMyBP-C antibodies p273, p282, p302, total cMyBP-C antibodies, and housekeeping protein antibodies alpha-actin or *HPRT*. Whole homogenates from left ventricular infarct tissue from MI rats and the same area from sham rats were used for Wes. Each lane represents one individual capillary loaded with the same amount of proteins. Original full length Wes images are presented in Supplementary Figs. S3–S5. (**B**) Quantification of cMyBP-C p273, p282, and p302 normalized to housekeeping proteins. Data represent means ± SEM (N = 6–8). **p < 0.01; ***p < 0.005; ****p < 0.001; unpaired two-tailed Student’s t-test.

To test the effect of cMyBP-C phosphorylation status on the MI-HF rat model, we collected protein samples from the left ventricular (LV) infarct zone of MI rats and a comparable area from sham animals at five different time points post-MI surgery: day 1, day 3, week 1, week 4 and week 8. Global cardiac functional parameters were measured at four time points (day 3, week 1, week 4 and week 8) post-MI surgery to confirm the development of heart failure (Supplementary Fig. S1). cMyBP-C phosphorylation was detected using antibodies specifically targeting serine phosphorylation sites at positions 273, 282 and 302, as well as antibody to detect total cMyBP-C expression levels (Fig. 1, Supplementary Figs. S2–7). Alpha-actin was used as a housekeeping protein for normalization on day 1 and day 3. HPRT was used as a housekeeping protein for normalization at week 1, week 4, and week 8 post-MI, owing to the significant reduction in alpha-actin levels at week 1 post-MI (Supplementary Fig. S8). The findings suggest that the expression levels of total cMyBP-C, along with the three phosphorylated serine residues (p273, p282 and p302), were reduced significantly at early time points (day 1 and day 3), followed by a trend in reduction at week 1 post-MI samples (Fig. 1, Supplementary Figs. S2–S5). Surprisingly, the expression of all three phosphorylation sites increased at later time points (week 4 and week 8 post-MI samples; Supplementary Fig. S2), except for p273 at week 8, possibly the result of a compensatory effect as HF progressed. We noticed that the expression of total cMyBP-C closely followed the trend of cMyBP-C phosphorylation changes (Fig. 1, Supplementary Fig. S2), indicating that the main effect of cardiac injury was on total cMyBP-C expression. Therefore, we calculated the ratio of cMyBP-C phosphosites (p273, p282, and p302) to total cMyBP-C expression (Supplementary Fig. S8). The phosphorylation of cMyBP-C sites (p273 & p282) to total cMyBP-C was unchanged at day-1 post-MI, but significantly increased on all three phosphosites (p273, p282 and p302) at day 3, week 1, week 4 and week 8 post-myocardial infarction compared to sham. In addition, we also tested the expression of Troponin T and Troponin I, which also showed a consistent reduction through all time points (Supplementary Fig. S9). Interestingly, reduced expression of the myosin heavy chain (MHC) was delayed and was observed only on day 3 post-MI. A quick compensation on MHC expression occurred with a dramatic increase at later time points (week 4 and week 8 post-MI; Supplementary Fig. S9).

### cMyBP-C phosphorylation was reduced in human HCM and DCM patients

**Figure 2:**
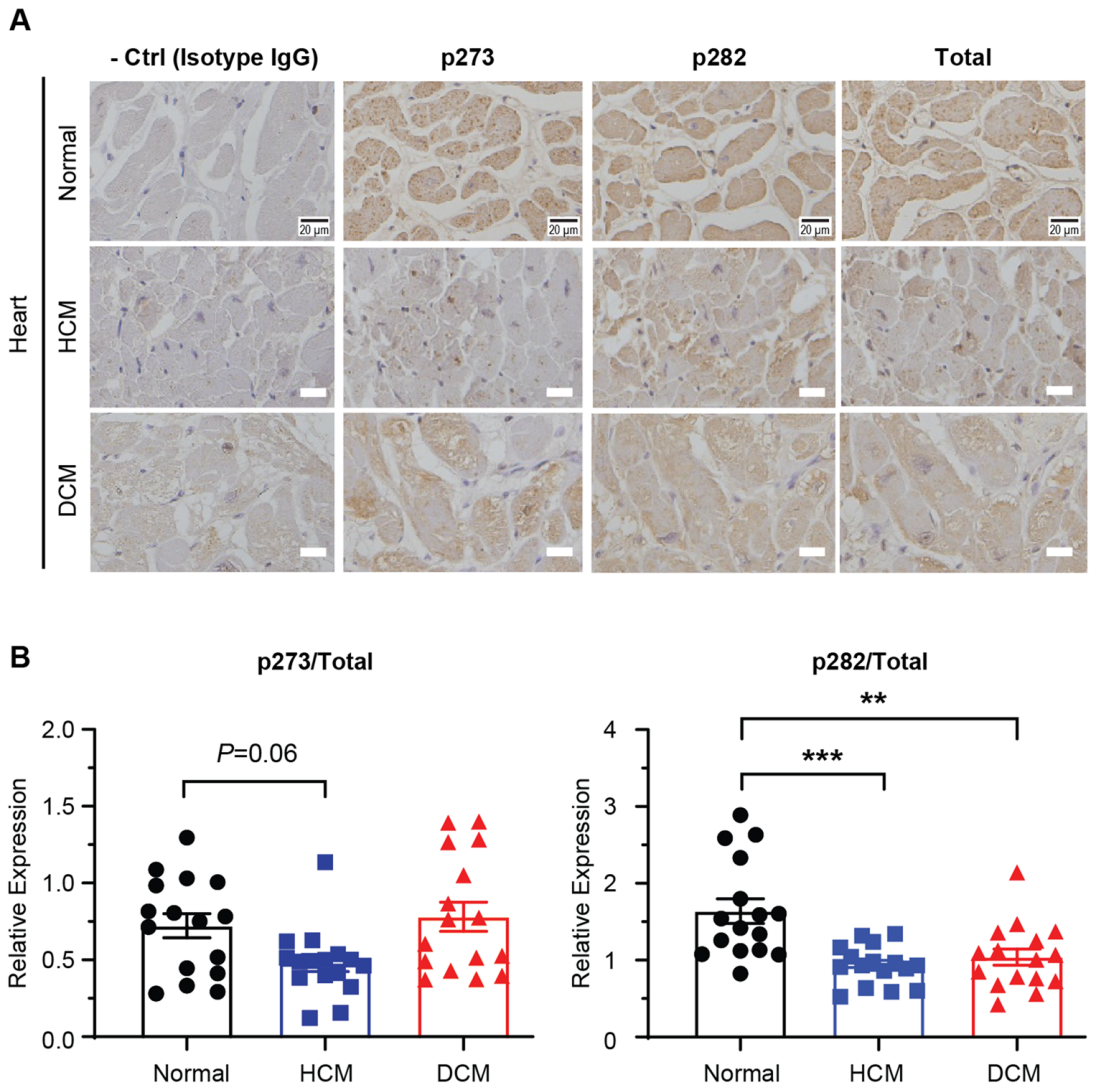
Immunohistochemistry of cardiac tissue sections from HCM and DCM patients showed reduced cMyBP-C phosphorylation compared to healthy donors. (**A**) IHC images of p273 and p282 in normal, HCM, and DCM heart samples. (**B**) Corresponding quantification of p273 and p282 in all heart samples. p273 and p282 values were normalized to total cMyBP-C values. Data represent mean ± SEM (N = 4 patients, n = 16 images). The scale bar in figures represents 20 µm. DCM, dilated cardiomyopathy; HCM, hypertrophic cardiomyopathy: IHC, immunohistochemistry. **p < 0.01; ***p < 0.005; one-way ANOVA with Tukey post-test.

To test the phosphorylation status of cMyBP-C in human HF, immunohistochemistry (IHC) was performed using cardiac tissue sections from normal healthy donors, as well as hypertrophic cardiomyopathy (HCM) and dilated cardiomyopathy (DCM) patients (N = 4 patients per group, Fig. 2A). HCM and DCM samples were confirmed by a trained physiologist. IHC was performed using antibodies against p273 and p282, as well as an antibody against total cMyBP-C. Relative expression of p273 was slightly reduced in cardiac tissues from HCM, but not from DCM patients (Normal 0.72 ± 0.08 *vs*. HCM 0.48 ± 0.06, p = 0.06; DCM 0.78 ± 0.10; n = 16; Fig. 2B). On the other hand, relative expression of p282 was reduced significantly in both HCM and DCM patients (Normal 1.64 ± 0.16 *vs*. HCM 0.93 ± 0.06, p < 0.005; Normal 1.64 ± 0.16 *vs*. DCM 1.04 ± 0.11, p < 0.01). Therefore, IHC data from human patients strongly supported reduced cMyBP-C phosphorylation in human HF.

### cMyBP-C peptide 302A or 302S did not affect ATPase activity in a recombinant steady-state ATPase assay

**Figure 3:**
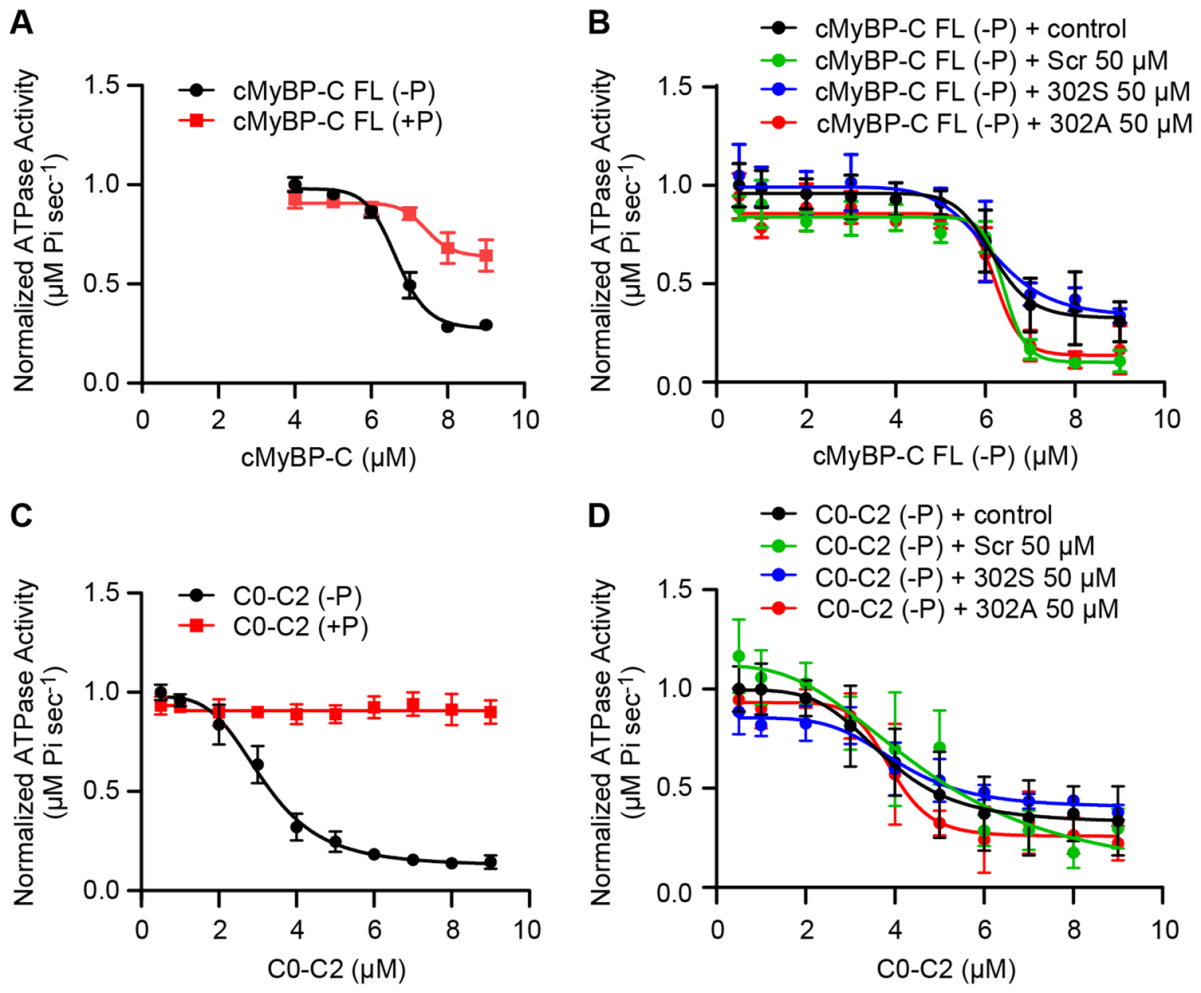
cMyBP-C peptides have no effect on steady-state ATPase assay with truncated cMyBP-C C0C2 or FL cMyBP-C. (**A**) Dephosphorylated FL cMyBP-C inhibited myosin ATPase activity more than inhibition of myosin by phosphorylated cMyBP-C. (**B**) cMyBP-C 302A or 302S peptides had no effect on myosin ATPase FL cMyBP-C activity. (**C**) Dephosphorylated cMyBP-C C0-C2 inhibited myosin ATPase activity, but not phosphorylated cMyBP-C. (**D**) cMyBP-C peptides 302A or 302S had no effect on CO-C2 myosin ATPase activity. Data represent mean ± SEM (N = 4–12). FL, full-length. Scr, scrambled.

The effects of cMyBP-C peptides 302A and 302S (Table 1) in modulating cMyBP-C was tested in three different in vitro assays. The steady-state ATPase assay involved myosin, actin and full-length (FL) cMyBP-C, or a truncated C0-C2 recombinant proteins (either unphosphorylated [−P] or phosphorylated [+P]). The ATPase activity was measured by detecting the Pi released from ATP hydrolysis. Inhibition of myosin ATPase activity was observed with increasing concentrations of both C0-C2 and FL cMyBP-C proteins (Fig. 3A,C). C0-C2 was much more potent in inhibiting ATPase activity when compared to FL cMyBP-C protein (3.16 ± 0.18 *vs*. 6.66 ± 0.11 µM). Although the phosphorylated protein, either C0-C2 or FL, shifted the inhibition curve to the right, neither 302A nor 302S peptide enhanced myosin ATPase activity tested at various concentrations (Fig. 3B,D). These results suggest that the recombinant sarcomere proteins myosin and actin alone in presence of the peptides may not be sufficient to impact ATPase activity to enable sarcomeric function. Instead, cMyBP-C peptides might only become functional and enhance ATPase activity in vitro in the presence of a more developed, intact sarcomere architecture.

**Table 1:** cMyBP-C peptides sequence and characterization.

Name	Peptide sequence	UV purity (%)	Observed [M+H]^+^ (Da)	Theoretical [M+H]^+^ (Da)	Mass accuracy
Scrambled	DALFKKAKSLRFELRSRD	96.4	2180.2241	2180.2298	2.61
302A	FSSLLKKRDAFRRDAKLE	93.5	2180.2202	2180.2298	4.4
302S	FSSLLKKRDSFRRDSKLE	90.7	2212.2175	2212.2197	0.99

cMyBP-C peptides were generated and characterized by high-resolution mass spectrometry. All peptide samples have purity values near or above 90% with accurate mass values in line with expectation based on the sequences.

### cMyBP-C peptides 302A and 302S did improve ATPase activity in myofibrils

**Figure 4:**
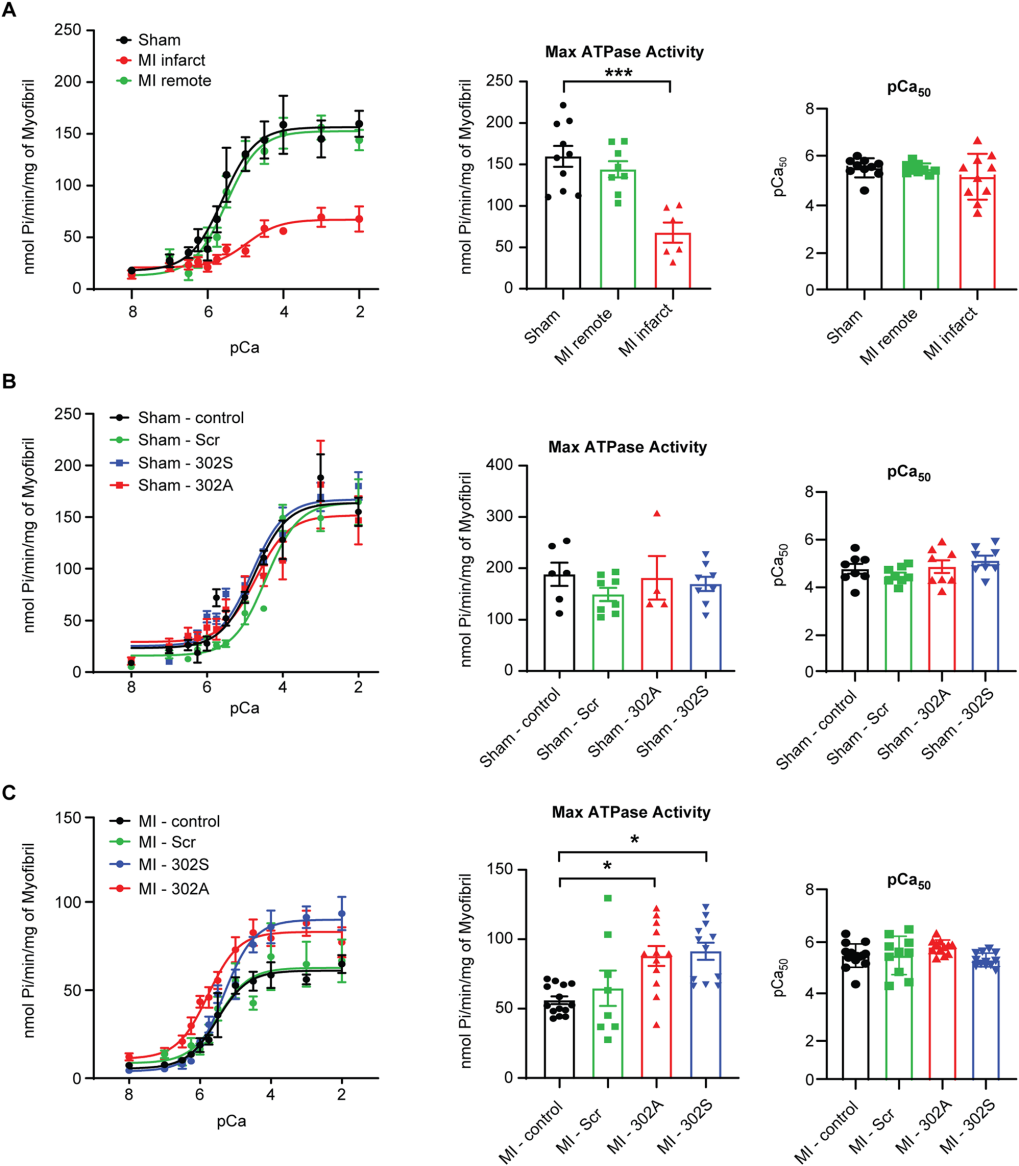
Myofibril ATPase activity was increased by cMyBP-C peptides 302A and 302S at week 1 in post-MI rats, but not in sham rats. (**A**) ATPase activity was reduced more than 50% in myofibrils from the MI infarct zone, but unchanged in myofibrils from the MI remote zone compared to myofibrils from sham hearts (left). Myofibril ATPase activity was normalized by myofibril protein used in the assay. The maximal ATPase activity at pCa 2.0 was plotted in the bar graph (middle). The pCa_50_ was also plotted to test calcium sensitivity (right). (**B**) cMyBP-C peptides 302A and 302S did not change maximal ATPase activity in myofibrils isolated from sham rats (left and middle). No change in calcium sensitivity was noted in any of the treatment groups in sham samples (right). (**C**) cMyBP-C peptides 302A and 302S increased maximal ATPase activity in myofibrils isolated from the infarct zone of week 1 post-MI rats compared to nontreatment control and scrambled peptide-treated myofibrils (left and middle). No change was noted in calcium sensitivity in any of the treatment groups in MI samples (right). Data represent mean ± SEM (N = 8–15); *p < 0.05; ***p < 0.005; one-way ANOVA with Tukey post-test. MI, myocardial infarction.

To further examine the role of cMyBP-C on ATPase activity with an intact sarcomere, we isolated myofibrils from heart samples of sham rats and week 1 post-MI rats in which sarcomere proteins are present in the native state. Using gel electrophoresis, integrity of the fully intact sarcomere was confirmed by verifying the presence of all key myofibrillar proteins, including MHC, cMyBP-C, actinin, troponin T, α-tropomyosin, as well as the myosin light chain (MLC) proteins (Supplementary Fig. S10A). We observed a consistent reduction in the phosphorylation of cMyBP-C p273, p282, and p302 in the myofibrils from week 1 post-MI compared to myofibrils isolated from sham animals (Supplementary Figs. S10B–D, S11), without changing the other major sarcomere proteins (Supplementary Fig. S10E,F). We then evaluated the effects of the cMyBP-C peptides in the myofibrillar ATPase assay, in which peptides can interact with intact sarcomere proteins (Fig. 4). A significant reduction in myosin ATPase activity was observed in myofibrillar proteins isolated from the MI infarct region compared to the myofibrillar proteins isolated from sham or MI-remote regions (sham 159.8 ± 12.59 *vs*. MI remote 144.1 ± 9.81 *vs*. MI infarct 67.8 ± 12.18, p < 0.005, Fig. 4A). Meanwhile, none of the peptides (scrambled, 302A or 302S) showed any effect on myofibrillar proteins from the sham hearts (Fig. 4B). Conversely, cMyBP-C peptides 302A and 302S peptides did improve maximal ATPase activity in myofibrils from infarcted hearts at week 1 post-MI from 60.9 ± 5.3 nmol Pi/min/mg (sham, n = 15) to 87.9 ± 7.1 nmol Pi/min/mg (302A, n = 12; *p* < 0.05) and 91.3 ± 6.2 nmol Pi/min/mg (302S, n = 12; *p* < 0.05), respectively (Fig. 4C).

**Figure 5:**
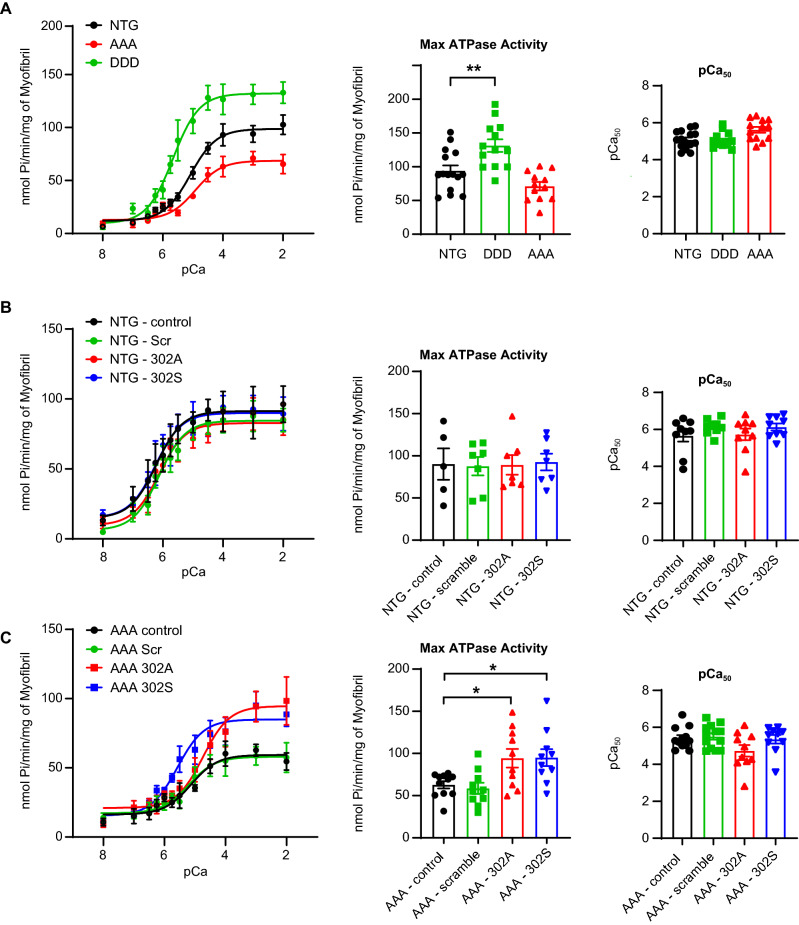
Effect of cMyBP-C peptides 302A and 302S in NTG and TG mice. (**A**) ATPase activity is higher in DDD TG mice compared to NTG mice. ATPase activity decreased in AAA TG mice (left). Maximal ATPase activity at pCa 2.0 was plotted in the bar graph (middle). The pCa_50_ was also plotted to test calcium sensitivity (right). (**B**) Neither cMyBP-C peptide 302A nor 302S changed maximal ATPase activity in myofibrils isolated from sham mice (left and middle). No change was noted in calcium sensitivity in any of the treatment groups in sham samples (right). (**C**) cMyBP-C peptides 302A and 302S increased maximal ATPase activity in myofibrils isolated from the transgenic mice compared to nontreatment control or scrambled peptide-treated myofibrils (left and middle). No change in calcium sensitivity was noted in any of the treatment groups in TG samples (right). Data represent mean ± SEM (N = 8–15); *p < 0.05; ***p < 0.005; one-way ANOVA with Tukey post-test. TG, transgenic mice.

Next, we investigated ATPase activity and the role of cMyBP-C in myofibrils isolated from cMyBP-C AAA and DDD transgenic mouse models (Fig. 5). Myosin ATPase activity was significantly upregulated in myofibrils from DDD mice (131.2 ± 9.33 nmol Pi/min/mg) compared to myofibrils from NTG (93.98 ± 7.85 nmol Pi/min/mg, p < 0.01) and AAA (71.06 ± 6.25 nmol Pi/min/mg) mice (Fig. 5A). Consistent with sham data, none of the peptides (scrambled, 302A and 302S) improved ATPase activity in myofibrils from NTG mice (Fig. 5B). On the other hand, both 302A and 302S peptides showed increased maximal ATPase activity from 62.74 ± 4.27 nmol Pi/min/mg (control, n = 11) and 58.58 ± 6.67 nmol Pi/min/mg (scrambled, n = 10) to 94.29 ± 11.01 nmol Pi/min/mg (302A, n = 10) and 95.05 ± 10.02 nmol Pi/min/mg (302S, n = 10), representing a 61% (302A) and 62% (302S) increase from scrambled, respectively (Fig. 5C). Neither cMyBP-C 302A nor 302S peptide affected calcium sensitivity in MI or AAA preclinical HF models.

### cMyBP-C peptide 302A and 302S improved papillary muscle force generation

**Figure 6:**
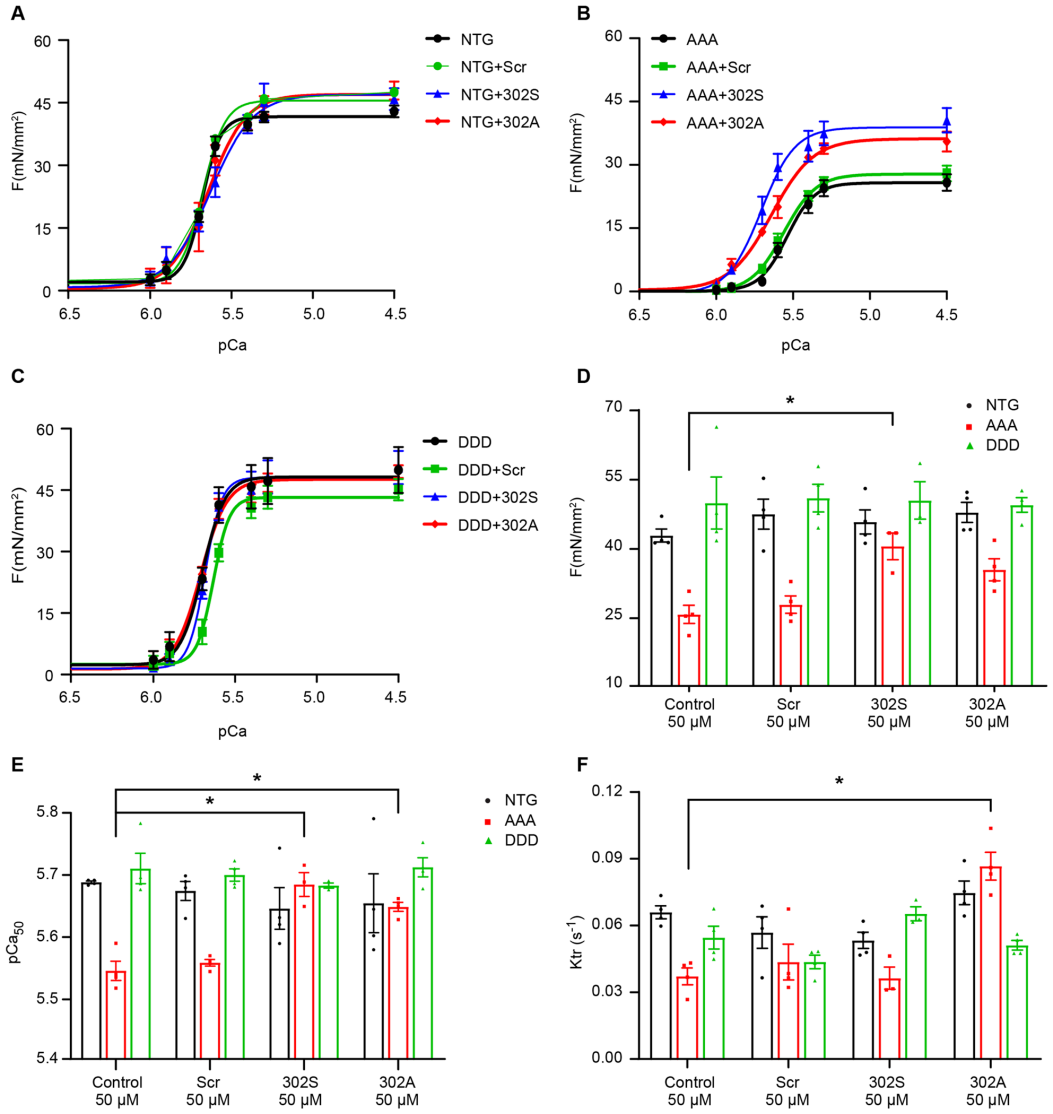
Cardiac function was improved by cMyBP-C peptides 302A and 302S in muscle fibers isolated from AAA transgenic mice, but not in NTG or DDD TG mice. Force pCa curves (**A**–**C**) generated at sarcomere length 2.0 μM in the presence of different peptides (50 μM). Maximum force production (**D**, mN/mm^2^), calcium sensitivity (**E**, pCa_50_), and rate of force regeneration (**F**, *k*_tr_ ) were measured in the presence of different peptides (50 μM) in NTG (white bar), AAA (red bar) and DDD (green bar). N = 4 papillary fibers per peptide treatment from 4 animals per group. *p < 0.05 one-way ANOVA with Tukey post-test. AAA, nonphosphorylated alanines; DDD, phospho-mimetic aspartic acids; NTG, non-transgenic mice. Scr, scrambled.

We extended the findings from myofibrils to investigate in papillary muscle which would be closer to the in vivo condition to determine if cMyBP-C synthetic peptides could impact sarcomere function. To accomplish this, pCa-force relationship was measured in papillary muscles from AAA and DDD mouse hearts at 3 months of age compared to age-matched NTG controls (Fig. 6). Compared to NTG and DDD fibers, we found that AAA fibers showed a significant deficit in maximal force development at pCa 4.5, as well as a significant decrease in calcium sensitivity, suggesting mechanical deficits at myofilament levels (Fig. 6A–E). Consistent with myofibril ATPase activity measurements, incubation with either 25 µM or 50 µM 302S peptide significantly improved force production in AAA papillary fibers, while 302A peptide also increased force, but not significantly (p = 0.0514) (Fig. 6D, Supplementary Fig. S12A). Both 302S and 302A peptides showed significant efficacy in improving calcium sensitivity in AAA fibers (Fig. 6E, Supplementary Fig. S12B). We also tested a phospho-mimetic cMyBP-C peptide 302D, which resulted in no improvement in force production in neither NTG, AAA, or DDD papillary fibers (Supplementary Fig. S13). To further characterize the effects of 302A and 302S peptides on cardiac function, we performed a force redevelopment rate (*k*_tr_) measurement as an indirect measure of relaxation. The 302A, but not 302S, peptide improved *k*_tr_ activity, indicating that the latter only affects contractility, not relaxation (Fig. 6F). The improved effects on *k*_tr_ were also observed with 302A peptide at 25 µM (Supplementary Fig. S12C).

## Discussion

cMyBP-C is a key regulator of cardiac contractility with more than 300 mutations in the *MYBPC3* gene directly linked to the development of cardiomyopathy and heart failure^22,23^. Although the phosphorylation of cMyBP-C has been shown to significantly decrease under pathological conditions in preclinical and clinical models, the dynamic change of cMyBP-C expression and its importance in cardiac kinetics at different time points have not been reported. In this study we characterized the dynamics of cMyBP-C dephosphorylation during the development of HF in a rat MI model. cMyBP-C phosphorylation decreased significantly within 1 week (on day 1 and day 3 post-MI, Fig. 1) after cardiac injury, but increased significantly at later time points (week 4 and week 8 post-MI, Supplementary Fig. S2), suggesting a compensatory effect in the heart. Total cMyBP-C also decreased at different time points in the MI preclinical HF model. These findings suggest that the cMyBP-C phosphorylation state is temporal and, when combined with decreased total cMyBP-C expression, contributes to the development of HF. Therefore, we hypothesized the utility of intervention strategies targeting cMyBP-C in its pathogenic dephosphorylated state at relevant time points based on our findings from preclinical animal models. The acute loss of total cMyBP-C in the bloodstream is consistent with previous studies, suggesting that cMyBP-C could serve as a potential diagnostic biomarker of MI and a prognostic marker of HF^24^. In human HCM and DCM patients, cMyBP-C phosphorylation is linked to the development of disease progression^25,26^. In HCM patients, both p273 and p282 were reduced (Fig. 2). The hierarchy of cMyBP-C phosphorylation sites has been reported^20^. Ser-282 has a unique regulatory role in that its phosphorylation is critical for the subsequent phosphorylation of Ser-302. Relevant to its importance in cMyBP-C function we did observe a decrease in ser-282 phosphorylation in both DCM and HCM patients (Fig. 2). Ser-273 is phosphorylated by both protein kinase A (PKA) and protein kinase C (PKC), unlike ser-282 which is primarily phosphorylated by PKA and CAMKII under non physiological calcium concentrations. In DCM patients, there is a possibility of compensation in PKA and/or PKC dynamics which could impact ser 273 phosphorylation. Hence this could have minimal effect in DCM and not HCM patients. Both ser-282 and ser-302 are involved in accelerating the kinetics of cMyBP-C and are phosphorylated by CAMKII. It has been shown that ablating ser282 phosphorylation decreases ser-302 phosphorylation by CAMKII. Although p302 was not examined owing to limited patient samples, our data indicated that different phosphorylation sites could be impacted in HCM and DCM patients.

Reduced phosphorylation of cMyBP-C has been linked to compromised contractility in heart failure patients^27^. Here, we used previously published cMyBP-C peptides 302A and 302S^21^, surrogates of the regulatory phosphorylation site serine 302, as a tool to determine whether modulating the dephosphorylation state of cMyBP-C can improve cardiac contraction and relaxation in experimental heart failure (HF) models. Therefore, to fully characterize the effects of peptides on modulating cMyBP-C, we evaluated the peptides in three different recombinant in vitro and *ex-vivo* assays: a steady state ATPase assay, a myofibril ATPase assay, and a papillary muscle assay. In the steady state ATPase assay, no change in ATPase activity was observed in any of the treatment groups (Fig. 3). However, when myofibril proteins were isolated from diseased models, i.e., the rat MI model or mouse cMyBP-C^AAA^ model, a consistent improvement of ATPase activity was observed in the 302S and 302A peptide-treated groups (Figs. 4, 5). This improvement in ATPase activity was consistent with the improvement of contractility using papillary muscle fibers isolated from cMyBP-C^AAA^ transgenic mice (Fig. 6). Together, these data showed the necessity of evaluating cMyBP-C–targeted therapies under pathological conditions. Additionally, the above findings strongly suggested the requirement of an intact sarcomere for the activity of peptides to properly capture cMyBP-C kinetics and its role in modulating cardiac function. Limited permeability of the peptides made immunolocalization of the peptides on intact cardiac myocytes or sarcomeres not feasible.

**Figure 7:**
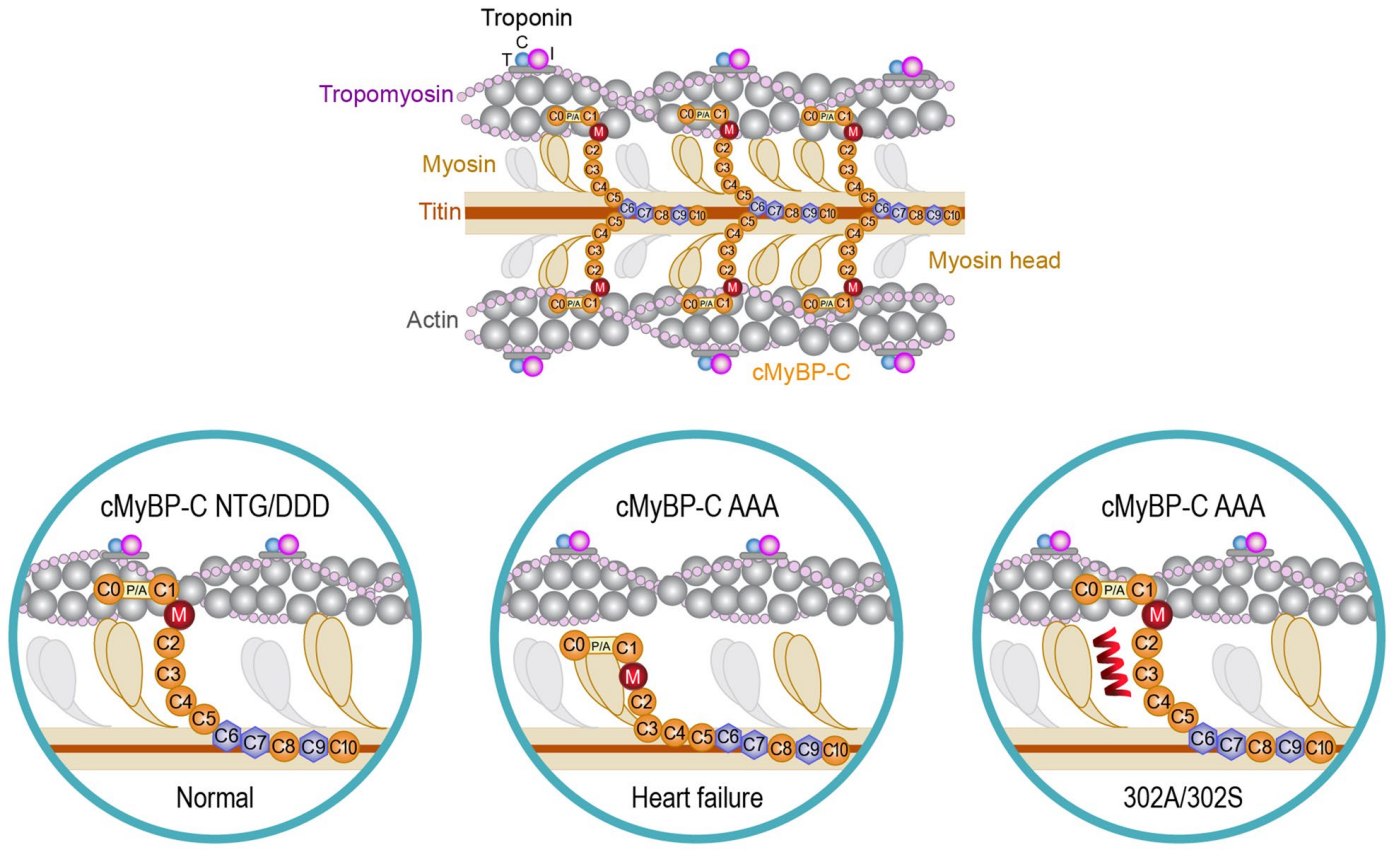
Diagram showing the effect of cMyBP-C phosphorylation state on actomyosin interaction vs. the effect of cMyBP-C peptides 302A and 302S in the context of cross-bridge formation. In the absence of phosphorylation (top), the position of the actin-binding site of cMyBP-C would lie about 3 nm from the thin filament. Phosphorylation of cMyBP-C (middle) would extend the cross-bridge to the surface of the thin filament and thereby loosen the packing of the rod portion of the myosin molecule. Permeabilizing 302A peptide inhibits myosin from interacting with cMyBP-C (bottom), resulting in its movement toward actin, which in turn results in the formation of actomyosin interactions and cross-bridges, thus enhancing contractility.

Since we observed the improved cardiac effects of peptides on HF models which had exhibited reduced cMyBP-C expression or phosphorylation, we hypothesized that the peptides probably interrupted the interaction of cMyBP-C and myosin, thus improving myosin head interaction with the thin filament and, hence, power stroke. Under physiological conditions (NTG/DDD or normal), phosphorylated cMyBP-C facilitates the activation of cross-bridge cycling, tethering the thick and thin sarcomeric filaments (Fig. 7; left panel). However, when cMyBP-C dephosphorylates in diseased conditions (AAA or HF), it strongly interacts with myosin, thus preventing its force-generating interaction with actin (Fig. 7; middle panel). The cMyBP-C peptides we tested could disrupt the tight interaction between cMyBP-C and myosin, releasing the thick filaments to resume their interaction with the thin filaments to improve muscle contractility (Fig. 7; right panel).

The relevance of cMyBP-C phosphorylation has been reported in multiple transgenic rodent models by substituting serine of the phosphorylation sites to mimic activated cMyBP-C, such as the DDD transgenic mouse model^18,19^. Reduction in phosphorylation sites in cMyBP-C can contribute to a decrease in protein expression. Here, we observed that such reduced phosphorylation correlated with reduced total cMyBP-C expression (Fig. 1). Recently, studies have reported on the delivery of *MYBPC3* cDNA to improve cardiac function. It has been reported that a single systemic dose of AAV9-carrying *MYBPC3* cDNA was able to restore *MYBPC3* mRNA and protein levels and prevent the development of left ventricular hypertrophy (LVH) in *MYBPC3*-targeted knock-in mice with only 20% cMyBP-C protein^28^. Another study reported that AAV6-carrying *MYBPC3* cDNA was able to achieve similar improvement in engineered 3D heart tissue^29,30^. In addition, AAV9-carrying *MYBPC3* cDNA has been shown to suppress the development of hypertrophy in human iPSC-derived cardiomyocytes from HCM patients^31^. However, sustained use of AAV9 as a therapy could have drawbacks by the lack of knowledge about cellular and whole-body disposition, dose-exposure relationship, and exposure–response relationship, as well as the effect of immunogenicity on these important properties^32^. Therefore, the search for specific molecules targeting cMyBP-C and its protein–protein interaction is critical to bring effective therapy from bench to bedside. Our study was limited by the poor permeability and stability of cMyBP-C peptides, restricting our assays to in vitro and ex vivo settings in which we used myofibril proteins and permeabilized papillary muscle fibers. Thus, further studies in an in vivo setting with permeable peptides and good stability would enable a more comprehensive understanding of the mechanism(s) of cMyBP-C modulation in cardiac function.

## Methodology

### Myocardial infarction rat model

The myocardial infarction (MI) rat model has been previously characterized^33^. Twenty-eight male Sprague–Dawley (SD) rats (Charles River Laboratories, Wilmington, MA), ranging from 8 to 10 weeks in age and weighing 180–250 g, were used to induce MI by left anterior descending coronary artery (LAD) ligation. We have followed the ARRIVE guidelines throughout the study for handling animals. All procedures were approved by the Institutional Ethics Committee for Use and Care of Laboratory Animals at Merck & Co., Inc., Kenilworth, NJ, USA. Briefly, the animals underwent left-sided thoracotomy under anesthesia. The pericardial sac was cut open, and LAD was identified. A coronary artery occluder was placed loosely around the LAD at ~ 2 mm below the origin, and then the occluder was pulled to ligate the coronary artery. Infarction was defined by blanching and discoloration of the left ventricle and ST elevation on the ECG. Then the chest was closed. The animals were closely monitored for surgical complications post-surgery. Twenty-three male SD rats with similar age and body weight were assigned as sham animals that received similar thoracotomy to access the heart and wound closure, but without LAD ligation. Post-MI evaluation to confirm infarcts was performed by echocardiography on day 3, week 1, week 4 and week 8. Meanwhile, the following global cardiac functional parameters were measured: LV end systolic and diastolic inner diameter (mm), LV end systolic and diastolic volume (µL), stroke volume (µL), cardiac output (mL/min), and heart rate (BPM) (Supplementary Fig. S1). Animals with small-sized infarct and ejection fraction (EF) > 40% were excluded from the study. The four termination time points were day 3, week 1, week 4, and week 8. At each termination time point, rats were anesthetized, and hearts were excised, snap-frozen in liquid nitrogen, and stored at − 80 °C until use.

### cMyBP-C transgenic mouse model

Previously described cardiac-specific transgenic phospho-ablated (AAA) and phospho-mimetic (DDD) mouse models of cMyBP-C with α-myosin heavy chain promoter were used to generate multiple lines of FVB/N mice^18,19,34,35^. In brief, the known key phosphorylation sites (Ser-273, Ser-282 and Ser-302) on cMyBP-C and two neighboring potentially alternative phosphorylation sites (Thr-272 and -281) were converted to alanine (A) and aspartic acid (D) residues using standard PCR-based methods. All experiments were conducted under institutional guidelines and approved by the University of Cincinnati Animal Care and Use Committee. Adult mice of mixed gender at 12–14 weeks of age were used in this study.

### cMyBP-C peptides

The cMyBP-C inhibitor peptides were synthesized at the research laboratories of Merck & Co., Inc., Kenilworth, NJ, USA. All peptides were characterized by high-resolution mass spectrometry to have confirmed purity values near, or above, 90% and accurate mass values in line with those expected based on the sequences. The sequences of the four original peptides used in this study are listed in Table 1^21^. Purity and accurate mass analysis were conducted simultaneously by reverse phase LC-UV/MS using a Waters ACQUITY I-class liquid chromatograph (Waters, Milford, MA) interfaced with a Xevo G2-XS QTOF mass spectrometer operating in the ESI + ionization mode. A Waters BEH C18, 1.7 μm, 130 Å, 2.1 × 150 mm stationary phase was used (part # 186003556) with 0.1% trifluoroacetic acid in distilled water (dH_2_O) (A) and 0.1% trifluoroacetic acid in acetonitrile (B) mobile phases. The column was held isothermal at 55 °C, with a linear gradient from 5 to 55% B over 25 min, using a 0.4 mL/min flow rate.

### Western immunoassay

The phosphorylation level of the cMyBP-C M-domain at three serine residues (p273, p282, p302) was determined in SD rats subjected to MI surgery using the Western Immunoassay (Wes) procedure (ProteinSimple, Santa Clara, CA). Left ventricle infarct tissue was lysed in a RIPA buffer (Thermo Fisher Scientific, Waltham, MA) supplemented with protease and phosphatase inhibitors. Protein estimation was performed as per kit (Pierce™ BCA Protein Assay Kit 23225, Thermo Fisher Scientific). Samples were prepared as per the Wes protocol, according to ProteinSimple recommendations. Antibodies used for the expression study are as follows: Anti-cMyBP-C p273 (1:250), cMyBP-C p-282 (1:250), cMyBP-C p302 (1:250) (all provided through material transfer agreement from Professor Sakthivel Sadayappan, College of Medicine, University of Cincinnati); cMyBP-C total (Santa Cruz Cat # SC-137182, Lot # E2913 [1:5 for Wes]); myosin heavy chain (Abcam ab207926 (3-48G5C7); alpha (cardiac) actin (Sigma-Aldrich A9357 clone AC1-20.4.2); Troponin I (Cell Signaling 13083S [D6F8]); and Troponin T (Sigma-Aldrich T6277 Clone JLT-12). Data were normalized to expressions of housekeeping proteins α-actin or hypoxanthine phosphoribosyl transferase (HPRT) to match the protein amount loaded.

### Immunohistochemistry

IHC studies were performed using cardiac serial tissue sections from adult human patients (normal subjects, HCM patients, and DCM patients). All methods were carried out in accordance with guidelines and regulations by Nanjing KeyGen BioTech Co., Ltd. and Nanjing GenScript Biotech Co., Ltd. All experimental protocols were performed in accordance with guidelines and regulations and approved by the Affiliated Drum Tower Hospital, Medical School of Nanjing University. Informed consent was obtained from all subjects and/or their legal guardian by the Affiliated Drum Tower Hospital, Medical School of Nanjing University. Briefly, tissue sections were deparaffinized in xylene and rehydrated through graded alcohol washes prior to incubating with a peroxidase block (3% H_2_O_2_-methanol) at room temperature for 10 min to inhibit endogenous peroxidase activity. After washing with phosphate-buffered saline (PBS), sections were blocked with 1% bovine serum albumin (BSA, 50-100μL) at room temperature for 1 h to prevent nonspecific binding of the detection reagent. After washing with PBS, sections were incubated with the primary antibody (50–100 μL; custom-made rabbit polyclonal antibodies [GenScript, Piscataway, NJ]): p-273 (AFRRTpSLAGAGC), p282 (GAGRRTpSDSHEDAC), and total protein (AFRRTSLAGAGC) in a humid chamber at 4 °C overnight. After washing, sections were then incubated with an enhancer (50–100 μL) at room temperature for 20 min and then washed with PBS. Sections were then incubated with ImmPRESS HRP PLUS Polymer (Mouse/Rabbit, Vector Laboratories, Burlingame, CA) at room temperature for 20 min and then washed with PBS. A mixture of 3,3′-Diaminobenzidine (DAB) chromogen and substrate was then added at room temperature for about 3–5 min to allow proper color development. The reaction was then terminated by washing the sections with water. Sections were then counterstained with Hematoxylin for 15 min and rinsed with water. Finally, sections were dehydrated in ascending graded alcohol rinse that ended with a xylene wash. Neutral balsam was used to cover the sections. The stained slides were imaged with a microscope at either 200× or 400× magnification to evaluate protein expression. The positive expression of phospho-proteins was determined by the ratio of the positive region to the total area and normalized using values of total protein expression.

### Recombinant steady-state ATPase assay

A steady-state ATPase assay was performed by measuring inorganic phosphate release using the colorimetric method. The ATPase reaction was performed in a reaction buffer consisting of 15 mM Tris–HCl, pH 7.5, 10 mM KCl, 2 mM MgCl_2_, and 0.1 mM EGTA. Myosin and FL cMyBP-C and C0-C2 domain were made at the research laboratories of Merck & Co., Inc., Kenilworth, NJ, USA, with modifications according to references^36,37^. Actin was made from rabbit skeletal muscle, with minor modifications, at the same research laboratories^38,39^. All proteins used in the assay were diluted in a reaction buffer. A 30 µL reaction was set up by adding FL or truncated cMyBP-C (C0-C2), myosin (1 µM), F-actin (10 µM) and ATP (2 mM). F-actin was prepared from G-actin by adding 50 mM KCl and 2 mM MgCl_2_, followed by brief vortexing and 30 min incubation on ice for polymerization. The plate was spun at 2000 rpm for 15 s to help reduce bubbles, and the reaction plate was incubated at 37 °C with constant shaking for 60 min. The reaction was stopped by diluting the proteins (1:20) in water. 30 µL of the 1:20 reaction mix were transferred to a fresh plate, and water was added to make up to 200 µL as per kit instructions (Abcam ab65622, Waltham, MA). Phosphate standards were also prepared as per kit instructions. Finally, 30 µL of detection reagent were added to all standard and sample wells, incubated at RT for 30 min with constant shaking, and then read at 650 nm endpoint in SpectraMax (Molecular Devices, San Jose, CA).

### Myofibrillar ATPase assay

Myofibrillar ATPase activity was determined by measuring inorganic phosphate release^40^. Myofibrillar proteins were isolated and purified, as previously reported^41,42^, and suspended in a F60 buffer with 500 mM NaCl. These proteins were extracted from week 1 post-MI and sham rat heart samples, as well as heart samples from cMyBP-C transgenic mice (NTG, AAA, and DDD). Protein was then determined by Coomassie blue staining (Bio-Rad Laboratories, Hercules, CA) of SDS-PAGE gels (Supplementary Fig. S10). The reaction mixture contained 0.25–0.5 mg/mL myofibrillar proteins, 15 mM Tris–HCl, 10 mM KCl, 2 mM MgCl_2_, 0.5 mM EGTA, and 5 mM ATP (pH 7.0). Assays were performed in a 96-well microtiter plate at pCa^2+^ concentrations (pCa) from 8.0 to 2.0 at 37 °C. The 120-µL reaction mixture was incubated at 37 °C for 15 min and centrifuged at 1500 rpm for 3 min. Each 30 µL of supernatant was transferred into a new 96-well microtiter plate and mixed with 170 µL distilled water (dH_2_O). A 30 µL detection reagent (Phosphate Assay Kit, Abcam) was added to create a reaction to develop color after which the production of inorganic phosphate was determined calorimetrically at 625 nm using a kinetic microplate reader (Molecular Devices). Calcium–stimulated ATPase activity was calculated by subtracting the activity at pCa 2.0 from the activity at pCa 8.0.

### Papillary muscle assay

Whole hearts were rapidly excised and washed with 1× Krebs–Henseleit Buffer (Sigma-Aldrich, St. Louis, MO), followed by the addition of 1 g d-glucose, 0.84 g NaHCO_3_, and 0.76 g BDM. Each heart was cut to carefully expose the papillary muscles in the LV. As previously described^34^, the papillary muscles were excised under a dissecting scope (V8 Stereo, PlanAPO S 0.63× FWD 81 mm, Zeiss, Dublin, CA) and permeabilized overnight in 1% Triton X-100 prediluted to 10% in mounting relaxing solution (97.92 mM KOH, 6.24 mM ATP, 10 mM EGTA, 10 mM Na2CrP, 47.58 mM potassium propionate, 100 mM BES, 6.54 mM MgCl_2_, and 1 mM DTT) at 4 °C to remove the cell membrane and membrane-bound proteins. The papillary muscles were then further trimmed into fiber bundles approximately 1 mm in length under the dissecting microscope. Straight fiber bundles were selected based on uniformity and were then attached at each end with aluminum t-clips. Each fiber bundle was gently washed with a fresh relaxing solution on ice and used within 12 h. The t-clipped fibers were then attached to a force transducer and a high-speed length controller (Aurora Scientific, Inc., Aurora, ON, Canada). Muscle dimensions (cross-sectional area, length) were determined using an ocular micrometer mounted on the dissection microscope (resolution, ~ 10 μM). These muscle dimensions were used to normalize contractile force and sarcomere length. The latter was set at 2.0 μM and was continuously monitored through Aurora’s High-Speed Video Sarcomere Length (HVSL) measurement system. The strength and rundown of the muscle fiber attachment was determined by exposing the attached fibers to the maximum calcium-saturating activating solution at the beginning and end of the experimental protocol. Developing isometric force was recorded at varying calcium concentrations (pCa from10.0 to 4.5) in the presence or absence of peptides, and zero baseline force level was subtracted from all force recordings at each activating cycle. All force measurements were corrected for rundown and normalized to the cross-sectional area. Fibers with more than 20% rundown were excluded from the data analysis. Data were acquired using Aurora’s 600A Real-Time Muscle Data Acquisition and Analysis System. Each individual force-calcium relationship was fitted to a modified Hill equation (Force/Forcemax = [Ca^2+^]n/(pCa_50_n + [Ca^2+^]n) in which n is the Hill slope. Similarly, the rate of tension redevelopment (*k*_tr_) was also measured in fibers harvested from AAA, DDD, and NTG heart tissue at pCa 4.5.

### Statistical analysis

All data are represented as the mean ± standard error of the mean (SEM), unless otherwise indicated. Statistical analysis was performed using GraphPad Prism (v 6.0, GraphPad Software, San Diego, CA). Data were analyzed using a two-way ANOVA with Tukey’s post-hoc test. A *p* < 0.05 value was considered to be statistically significant.

## Supplementary Information

Supplementary Figures.

## Abbreviations

AAA: Nonphosphorylated alanines
DCM: Dilated cardiomyopathy
DDD: Phospho-mimetic aspartic acids
dH_2_O: Distilled water
EDV: End diastolic volume
EF: Ejection fraction
ESV: End systolic volume
F: Force
FITC: Fluorescein isothiocyanate
FL: Full-length
HCM: Hypertrophic cardiomyopathy
*HPRT*: Hypoxanthine phosphoribosyl transferase gene
iPSC: Human-induced pluripotent stem cells
*k*_tr_: Force redevelopment rate
LVIDd: Left ventricular internal diameter end diastole
LVIDs: Left ventricular internal diameter end systole
MHC: Myosin heavy chain
MI: Myocardial infarction
MLC: Myosin light chain
NTG: Non-transgenic
α-TM: α-Tropomyosin
TnT: Troponin T
TnI: Troponin I
