# Supplementary material for: Modulation of myosin by cardiac myosin binding protein-C peptides improves cardiac contractility in ex-vivo experimental heart failure models
Source: Sci Rep. 2022 Mar 14;12:4337. doi: 10.1038/s41598-022-08169-1 (PMC8921245; doi:10.1038/s41598-022-08169-1)
Supplement: Supplementary file 1 — Supplementary Figures. [file 41598_2022_8169_MOESM1_ESM.pdf]

Supplemental Fig S1

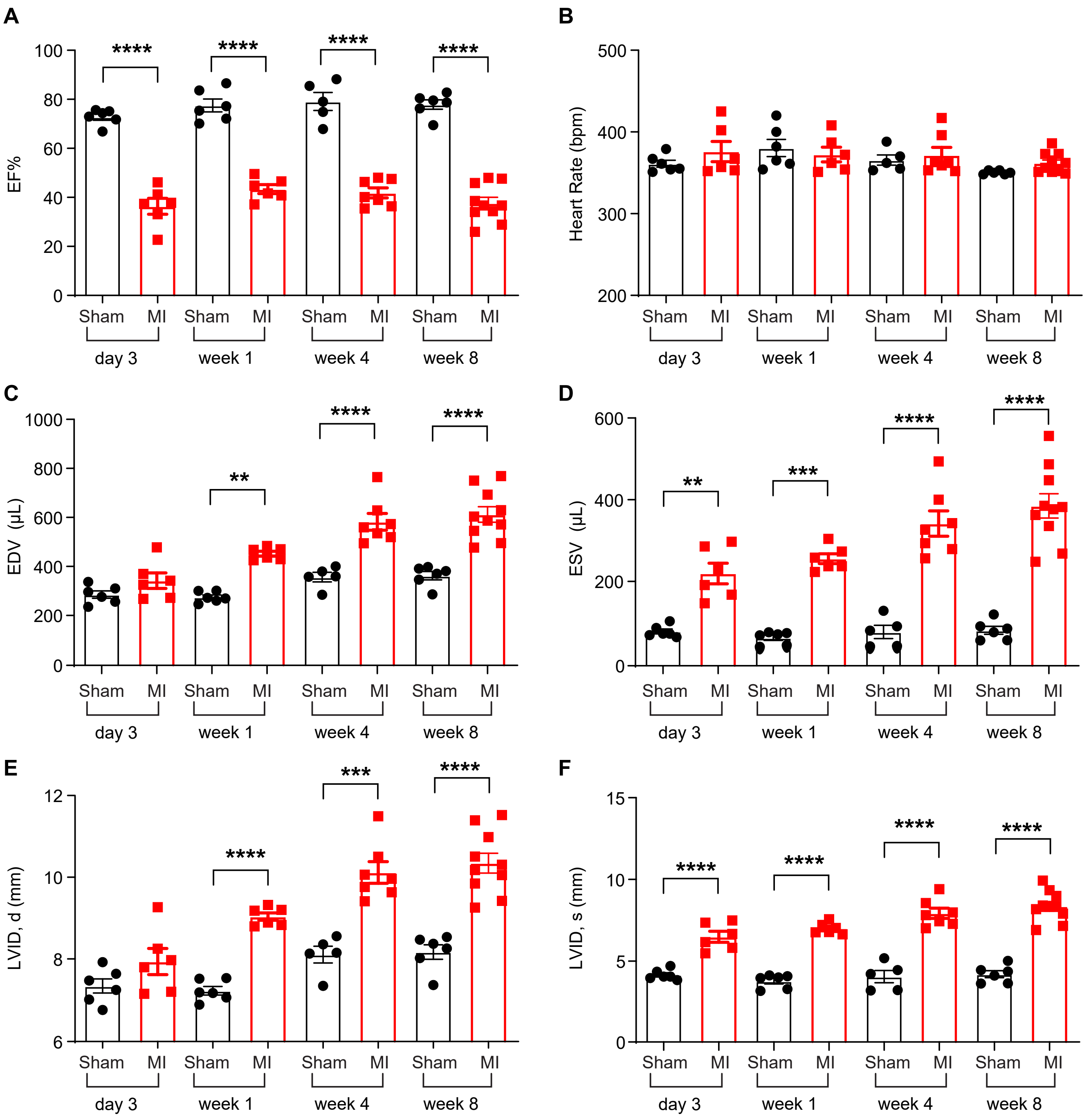

Supplementary Fig. S1. Echocardiographic evaluation of compromised cardiac function was observed in rats at day 3, week 1, week 4 and week 8 post-MI. (A) Consistent ejection fraction (%) reduction was observed in all time points (day 3, week 1, week 4 and week 8). (B) Heart rate remained unchanged in all time points (C) EDV was significantly increased in week 1, week 4 and week 8 but not in day 3 post-MI. (D) ESV was significantly increased in day 3, week 1, week 4 and week 8 post-MI. (E) LVIDd was increased at all time points after day 3 post-MI. (F) LVIDs was significantly increased in all time points (day 3, week 1, week 4 and week 8). Data represent mean ± SEM (N=5-10). EF, ejection fraction; EDV, end diastolic volume; ESV, end systolic volume; LVIDd, left ventricular internal diameter end diastole; LVIDs, left ventricular internal diameter end systole; MI, myocardial infarction. *p<0.05; **p<0.01; ***p<0.005; ****p<0.001; one-way ANOVA with Tukey post-test.

Supplemental Fig S2

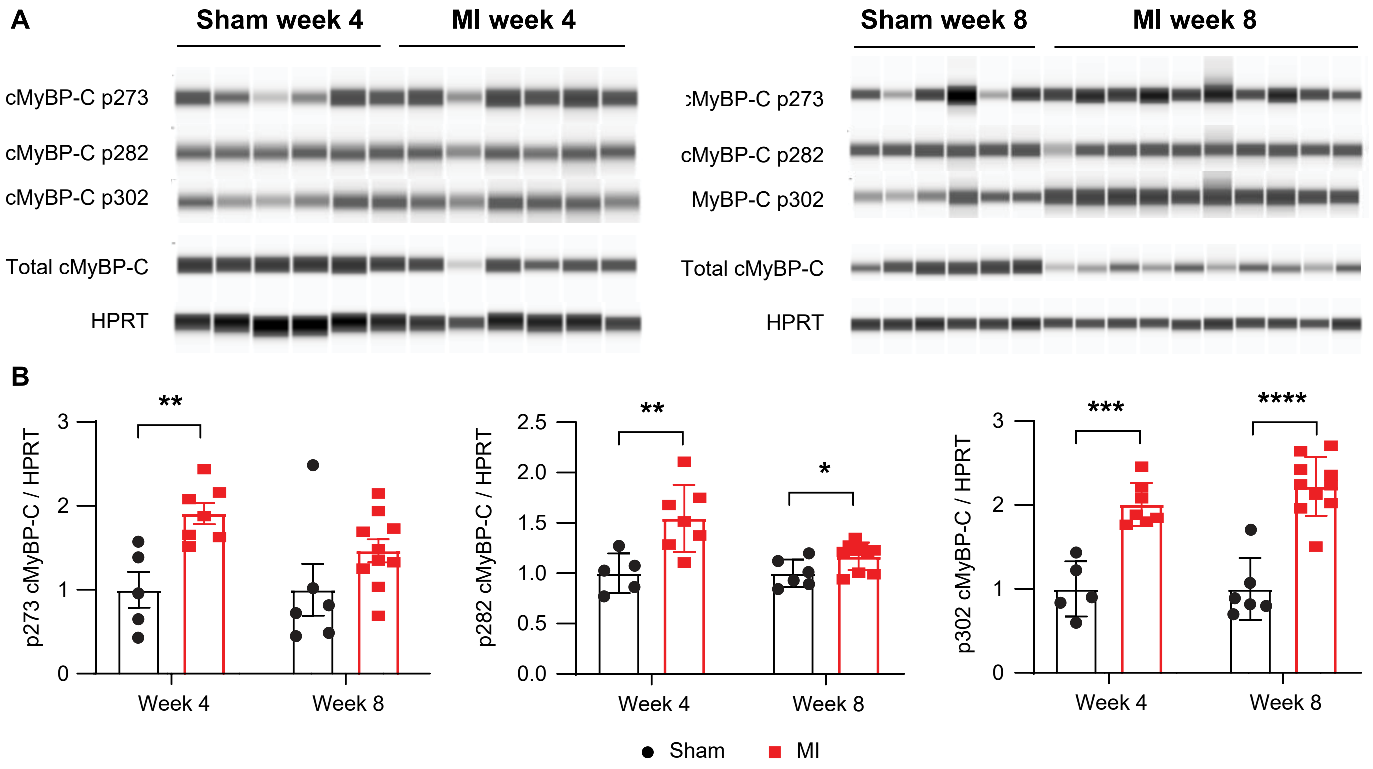

Supplementary Fig. S2. cMyBP-C phosphorylation was increased in rats at week 4 & week 8 post-MI compared to sham. (A) Western blot (Wes) analysis of cMyBP-C p273, p282, p302, total cMyBP-C, and housekeeping protein HPRT. Original full length Wes images are presented in Supplementary Fig. S6 and S7 at the same exposure level. (B) Quantification of p273, p282, and p302 to housekeeping protein HPRT. Data represent mean ± SEM (N=6-10). *p<0.05; **p<0.01; ***p<0.005; ****p<0.001; one-way ANOVA with Tukey post-test.

Supplemental Fig S3

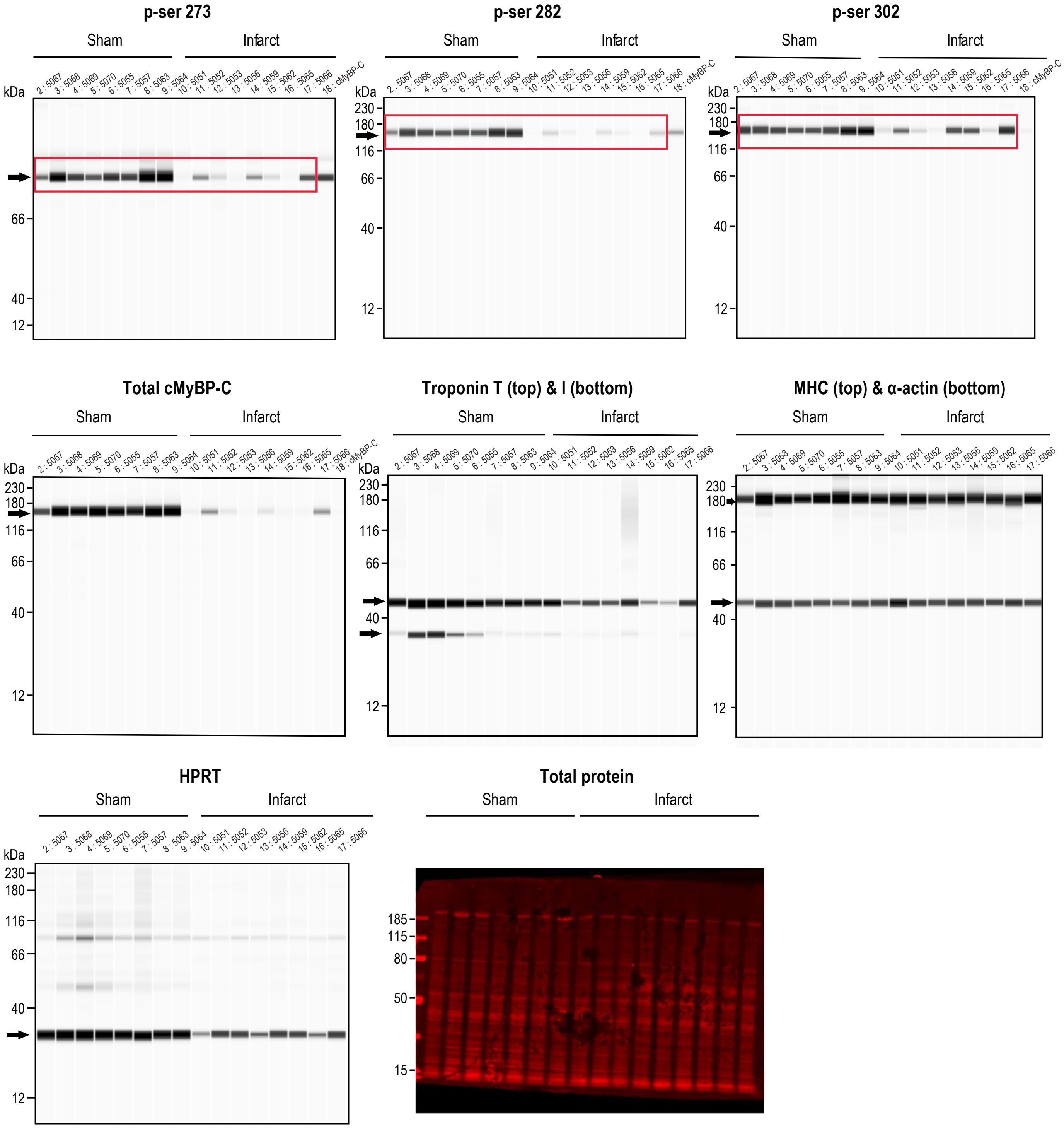

Supplementary Fig. S3. Day 1 post MI western blot (Wes) analysis of cMyBP-C p273, p282, p302, total cMyBP-C, troponin T, troponin I, myosin heavy chain (MHC), and housekeeping proteins α-actin and HPRT. cMyBP-C phosphorylation and total protein level was decreased in rats at day 1 post MI compared to sham. Red box: gel images shown in Fig. 1.

Supplemental Fig S4

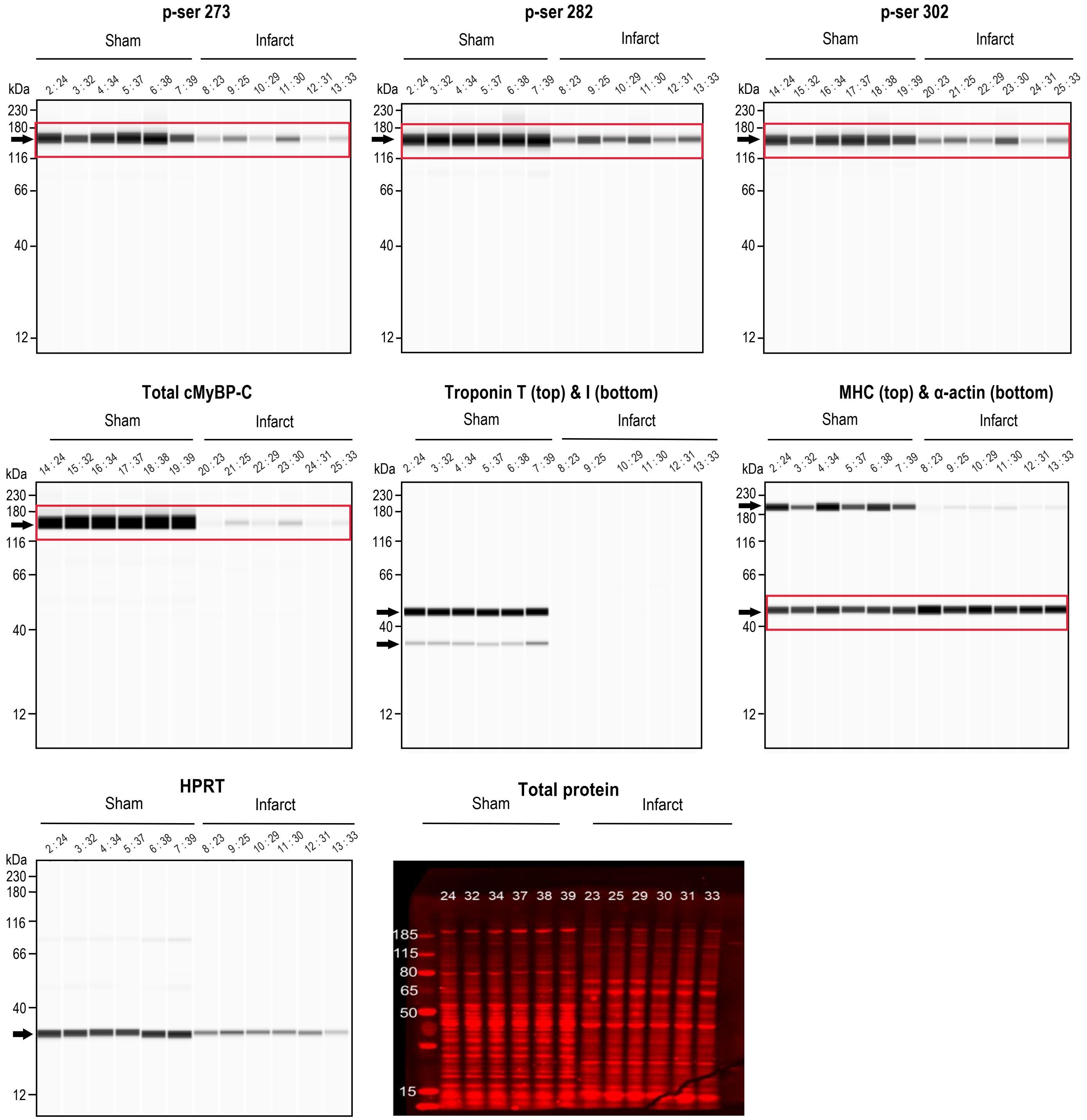

Supplementary Fig. S4. Day 3 post MI western blot (Wes) analysis of cMyBP-C p273, p282, p302, total cMyBP-C, troponin T, troponin I, myosin heavy chain (MHC), and housekeeping proteins α-actin and HPRT. cMyBP-C phosphorylation and total protein level was decreased in rats at 3 day post MI compared to sham. Red box: gel images shown in Fig. 1.

Supplemental Fig S5

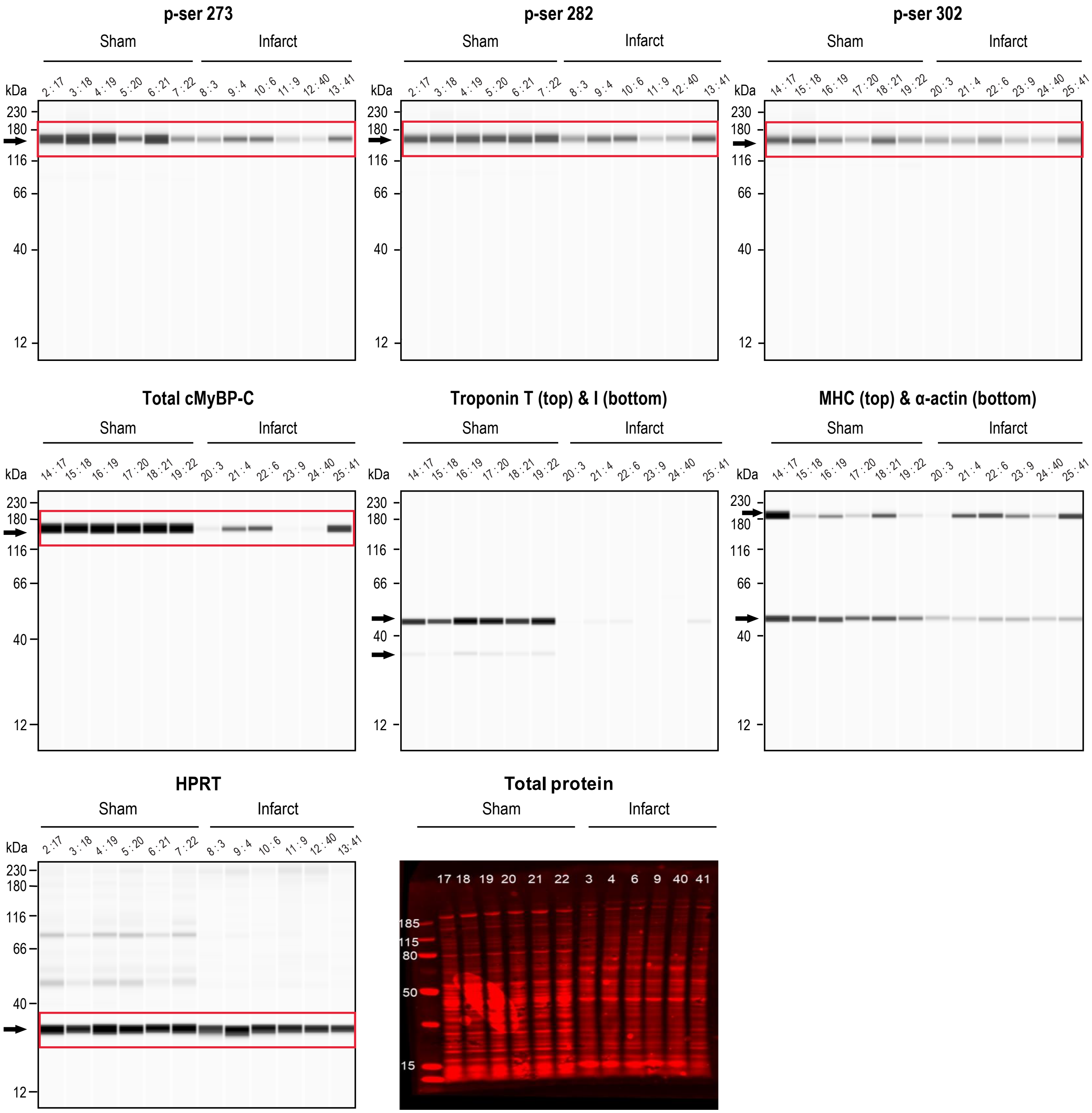

Supplementary Fig. S5. Week 1 post MI western blot (Wes) analysis of cMyBP-C p273, p282, p302, total cMyBP-C, troponin T, troponin I, myosin heavy chain (MHC), and housekeeping proteins α-actin and HPRT. cMyBP-C phosphorylation and total protein level was decreased in rats at week 1 post MI compared to sham. Red box: gel images shown in Fig. 1.

Supplemental Fig S6

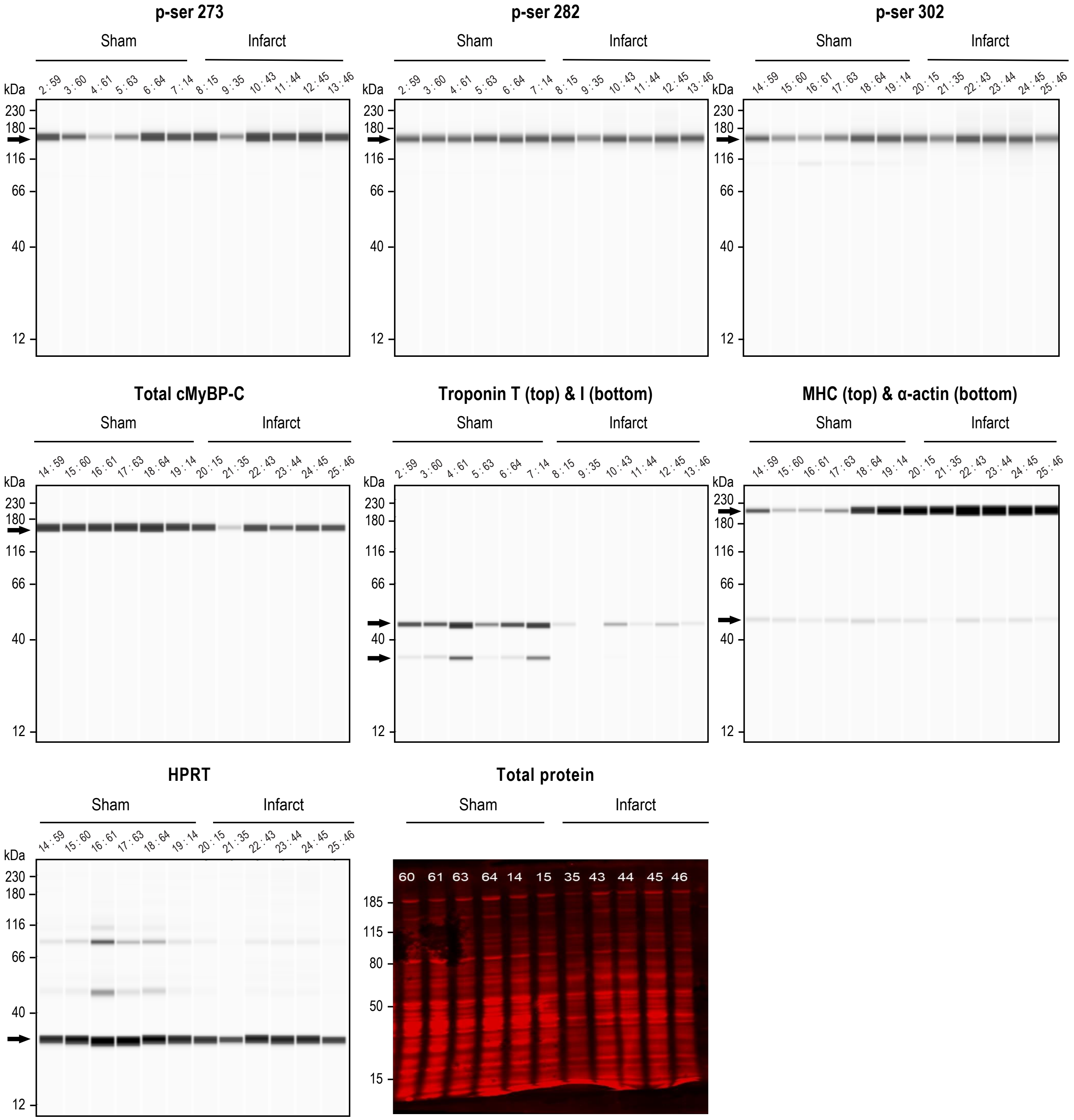

Supplementary Fig. S6. Week 4 post MI western blot (Wes) analysis of cMyBP-C p273, p282, p302, total cMyBP-C, troponin T, troponin I, myosin heavy chain (MHC), and housekeeping proteins α-actin and HPRT. cMyBP-C phosphorylation and total protein level was decreased in rats at week 4 post MI compared to sham.

Supplemental Fig S7

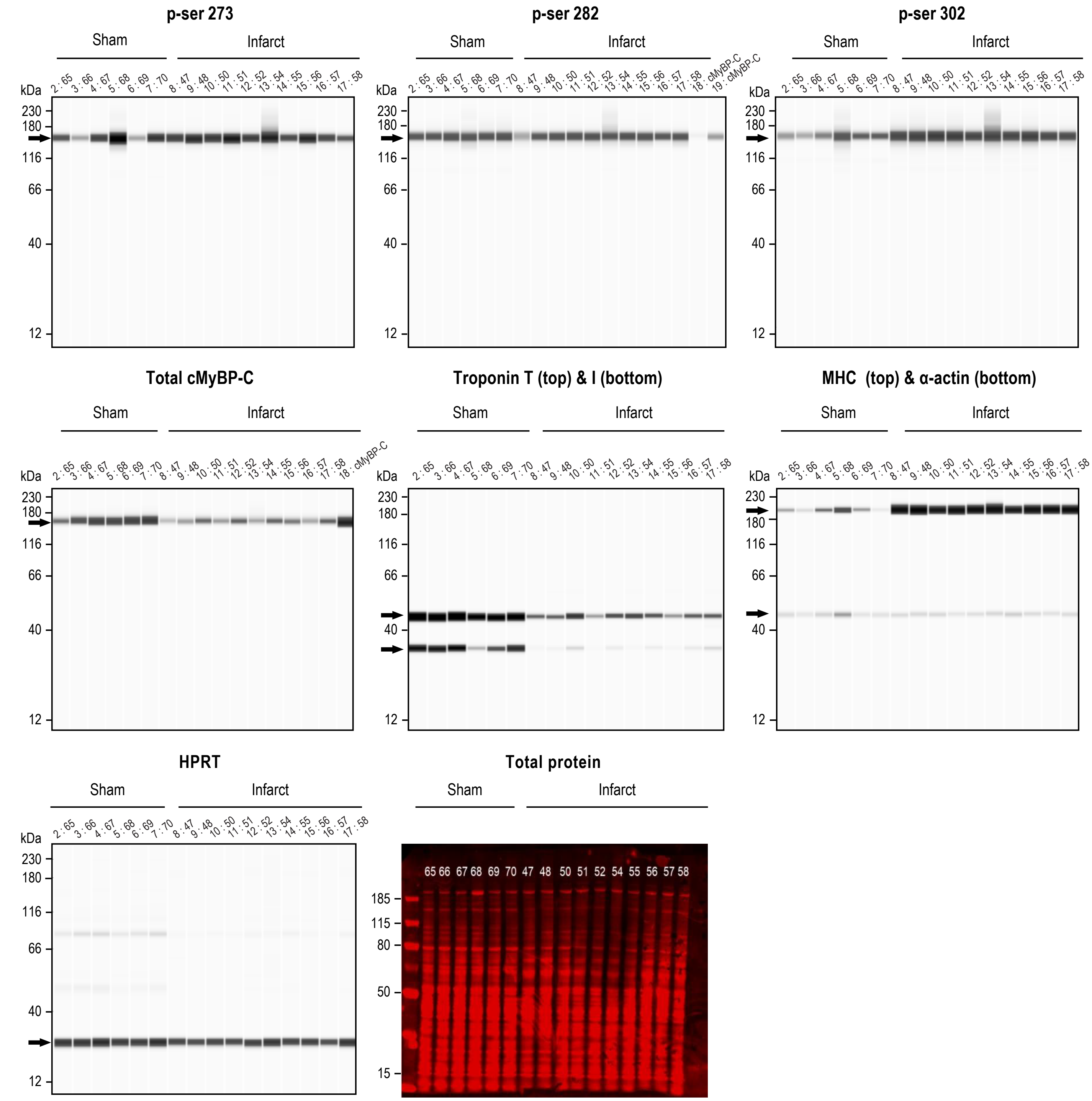

Supplementary Fig. S7. Week 8 post MI western blot (Wes) analysis of cMyBP-C p273, p282, p302, total cMyBP-C, troponin T, troponin I, myosin heavy chain (MHC), and housekeeping proteins α-actin and HPRT. cMyBP-C phosphorylation and total protein level was decreased in rats at week 8 post MI compared to sham.

Supplemental Fig S8

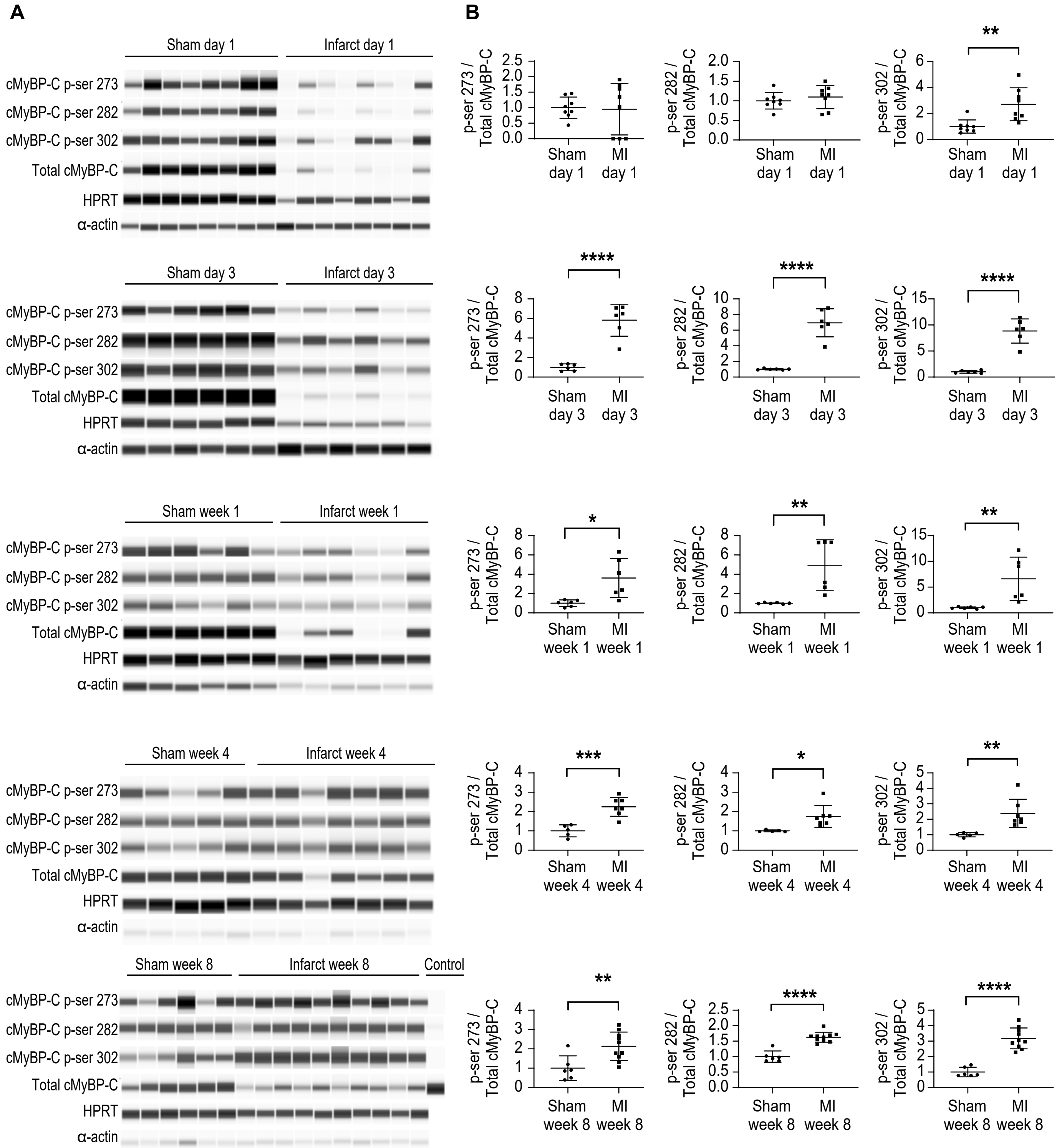

Supplementary Fig. S8. cMyBP-C phosphorylation normalized to total protein showed gradual increase in expression level on day 1, day 3, week 1, week 4 and week 8 post-MI. (A) Representative Wes images of cMyBP-C p273, p282, p302, total cMyBP-C, and house keeping genes HPRT and α-actin; Original full length Wes images are presented in Supplementary Fig. S3-S7 at the same exposure level. **(B)** Quantification of cMyBP-C p-273, p-282, and p-302 to total cMyBP-C. *p<0.05; **p<0.01; ***p<0.005; ****p<0.001; one-way ANOVA with Tukey post-test.

Supplemental Fig S9

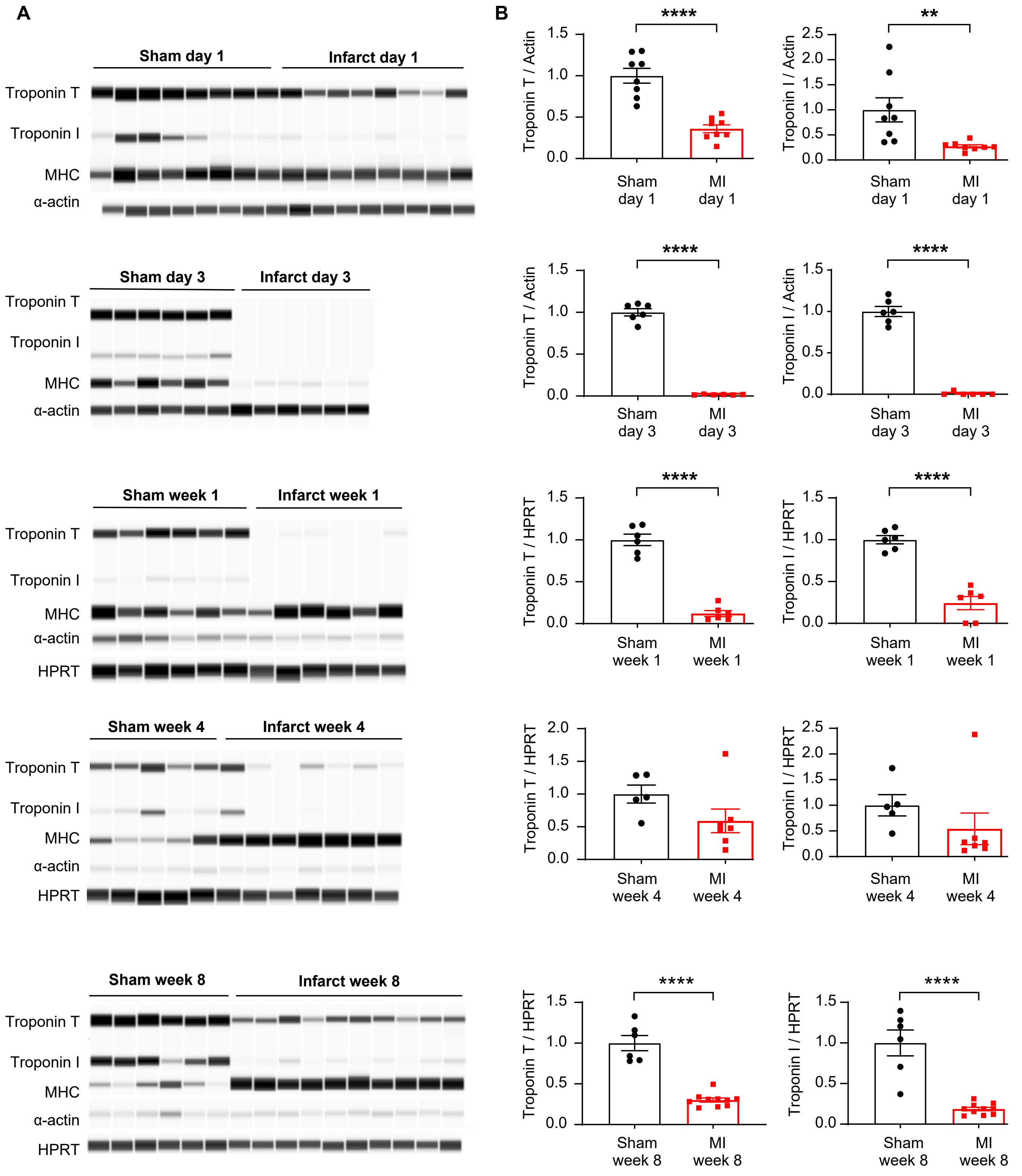

Supplementary Fig. S9. Expression of Troponin T and Troponin I was significantly reduced at day 1, day 3, week 1, week 4 and week 8 post-MI. (A) Western blot (Wes) analysis of Troponin T, Troponin I, MHC and housekeeping genes α-actin and HRPT. Original full length Wes images are presented in Supplementary Fig. S3-S7 at the same exposure level. (B) Quantification of Troponin T and Troponin I expression normalized to housekeeping protein showed reduction at day 1, day 3, week 1, and week 8 post-MI. Data represent mean ± SEM (N=5-10). **p<0.01; ****p<0.001; one-way ANOVA with Tukey post-test. MHC, myosin heavy chain.

Supplemental Fig S10

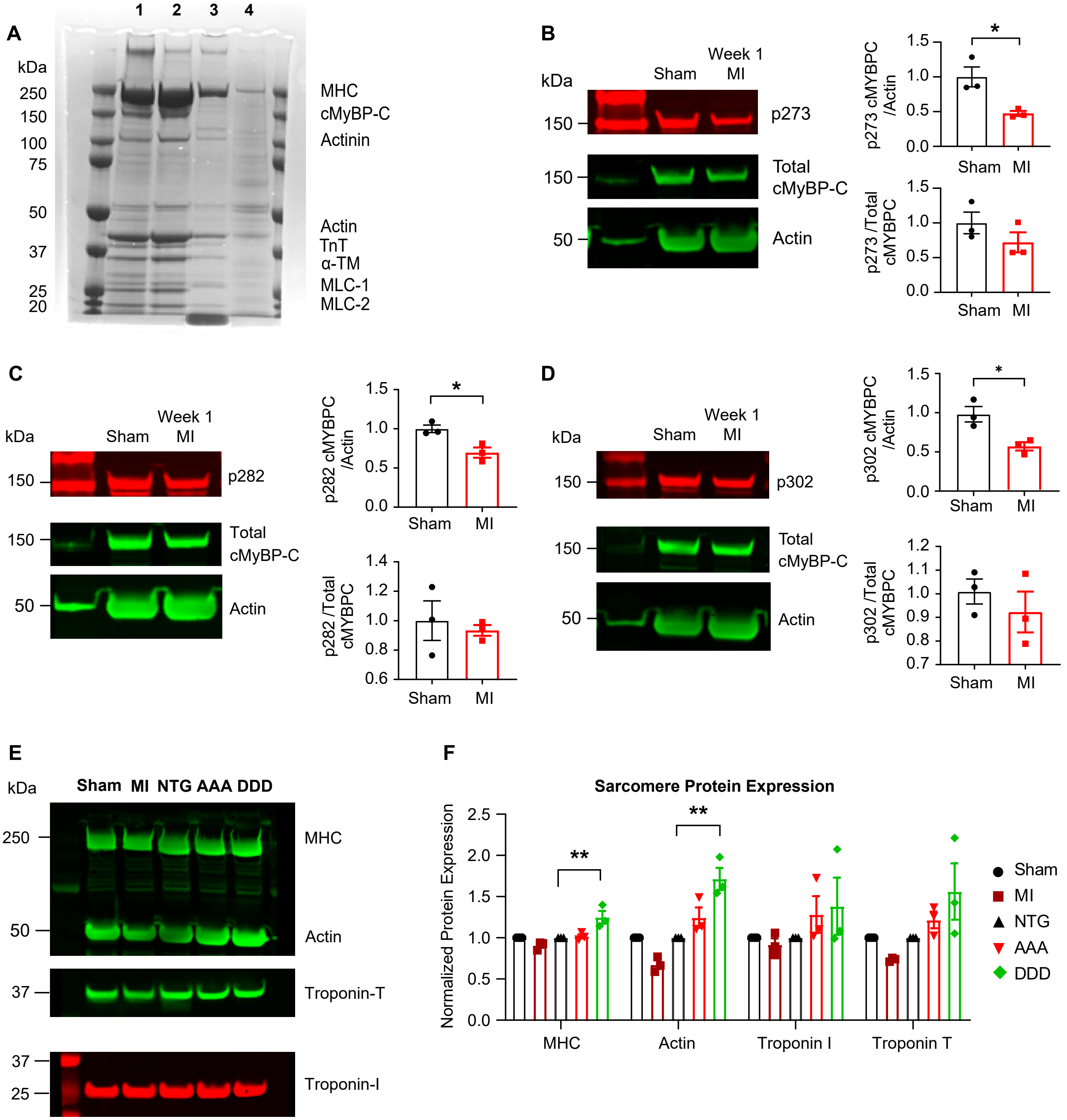

Supplementary Fig. S10. cMyBP-C phosphorylation was reduced but major sarcomere proteins did not change in myofibril samples from HF models (week 1 post-MI rats and AAA mice). (A) Coomassie Blue staining of sarcomere proteins in isolated myofibrils from sham rats. 4 conditions have been tested: 1 - Reaction buffer 1 + high salt (500 mM); 2- Reaction buffer 1 + Urea; 3 - Reaction buffer 2 + Urea; 4 - Reaction buffer 2 only. (B-D) Representative traditional western blot analysis using site-specific phospho-cMyBP-C antibodies p273, p282, and p302 in sham and MI myofibrils. Quantification was performed using either housekeeping protein (actin), or total cMyBP-C. Data represent mean ± SEM (N=3). *p<0.05; unpaired two-tailed Student's t-test. MI, myocardial infarction. (E) Western blot analysis of MHC, actin, troponin I and troponin T. (F) Quantification of major sarcomere proteins in sham, MI, NTG, AAA and DDD mice. The expression of MHC and actin was higher in DDD mice than in NTG mice. Original full length western blot images are presented in Supplementary Fig. S11 at the same exposure level. Data represent mean ± SEM (N=3). **p<0.01; one-way ANOVA with Tukey post-test. AAA, nonphosphorylated alanines; DDD, phospho-mimetic aspartic acids; MHC, myosin heavy chain; MI, myocardial infarction; NTG, non-transgenic mice.

Supplemental Fig S11

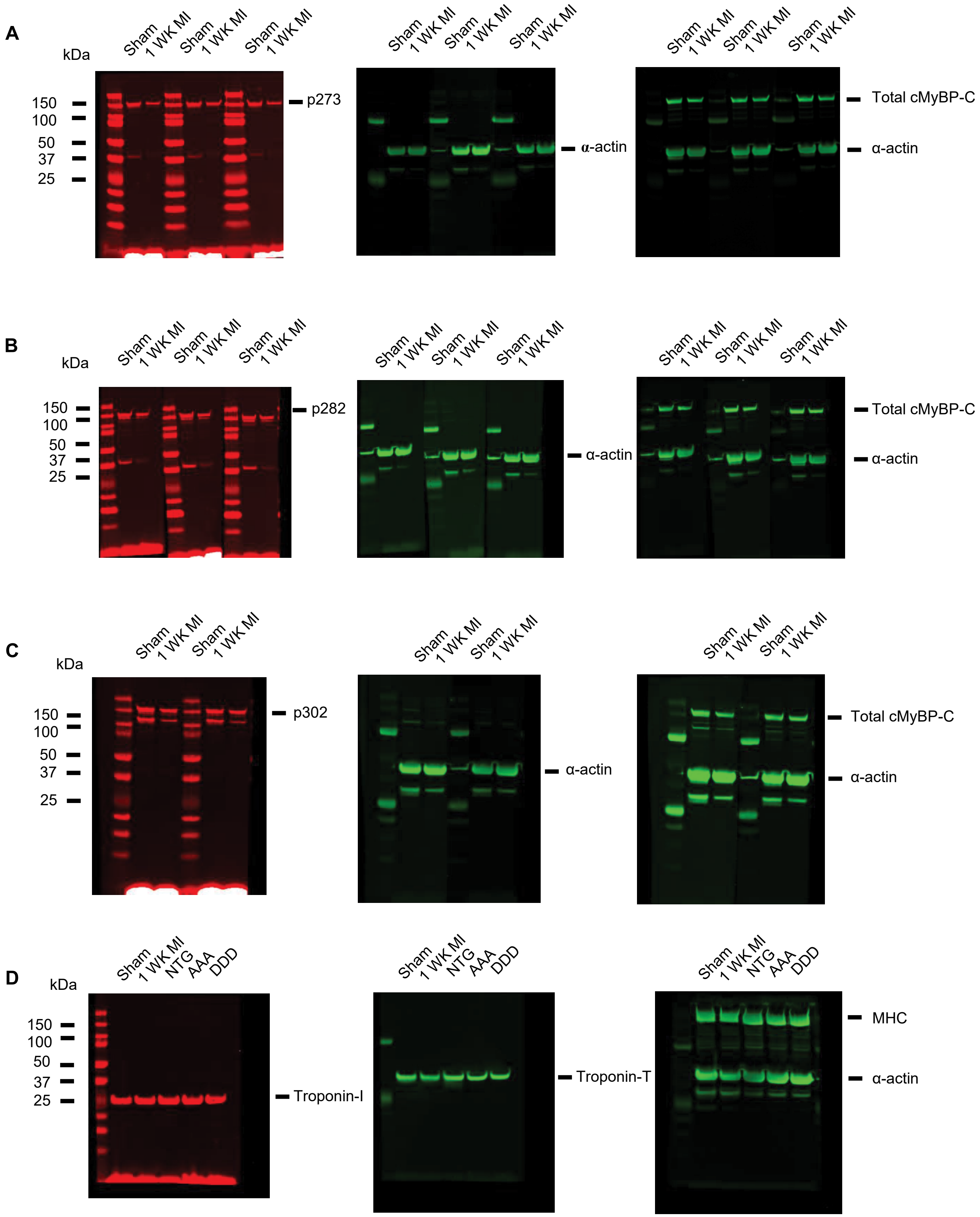

Supplementary Fig. S11. cMyBP-C phosphorylation and total protein level was decreased in myofibrils from week 1 post MI without changing other sarcomere protein levels. Traditional western blot gel images showed that the expression of p273 (**A**), p282 (**B**) and p302 (**C**) were decreased in myofibrils from week 1 post MI rats comparing to myofibrils from sham rats, and myosin heavy chain (MHC), troponin-T and troponin-I was not changed in MI or AAA (**D**).

Supplemental Fig S12

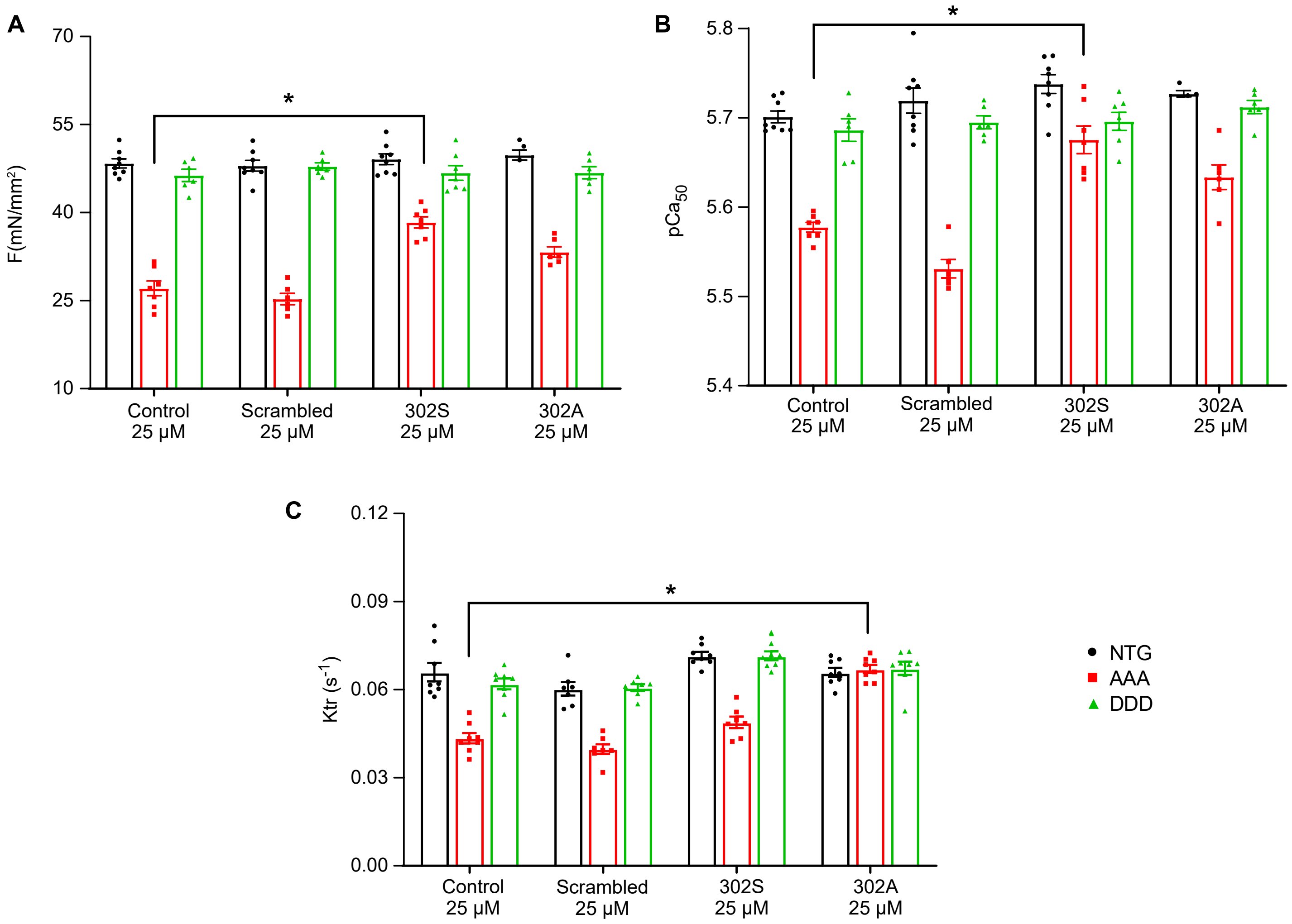

Supplementary Fig. S12. Low Dose (25 uM) of cMyBP-C peptides on AAA, DDD, and NTG muscle fiber mechanics. Maximal force generated (A), calcium sensitivity (B), and rate of force regeneration (C) at sarcomere length 2.0 µM in the presence of different peptide concentrations (25, 50 and 100 µM). *$p<0.05$ one-way ANOVA with Tukey post-test. AAA, nonphosphorylated alanines; DDD, phospho-mimetic aspartic acids; NTG, non-transgenic mice.

Supplemental Fig S13

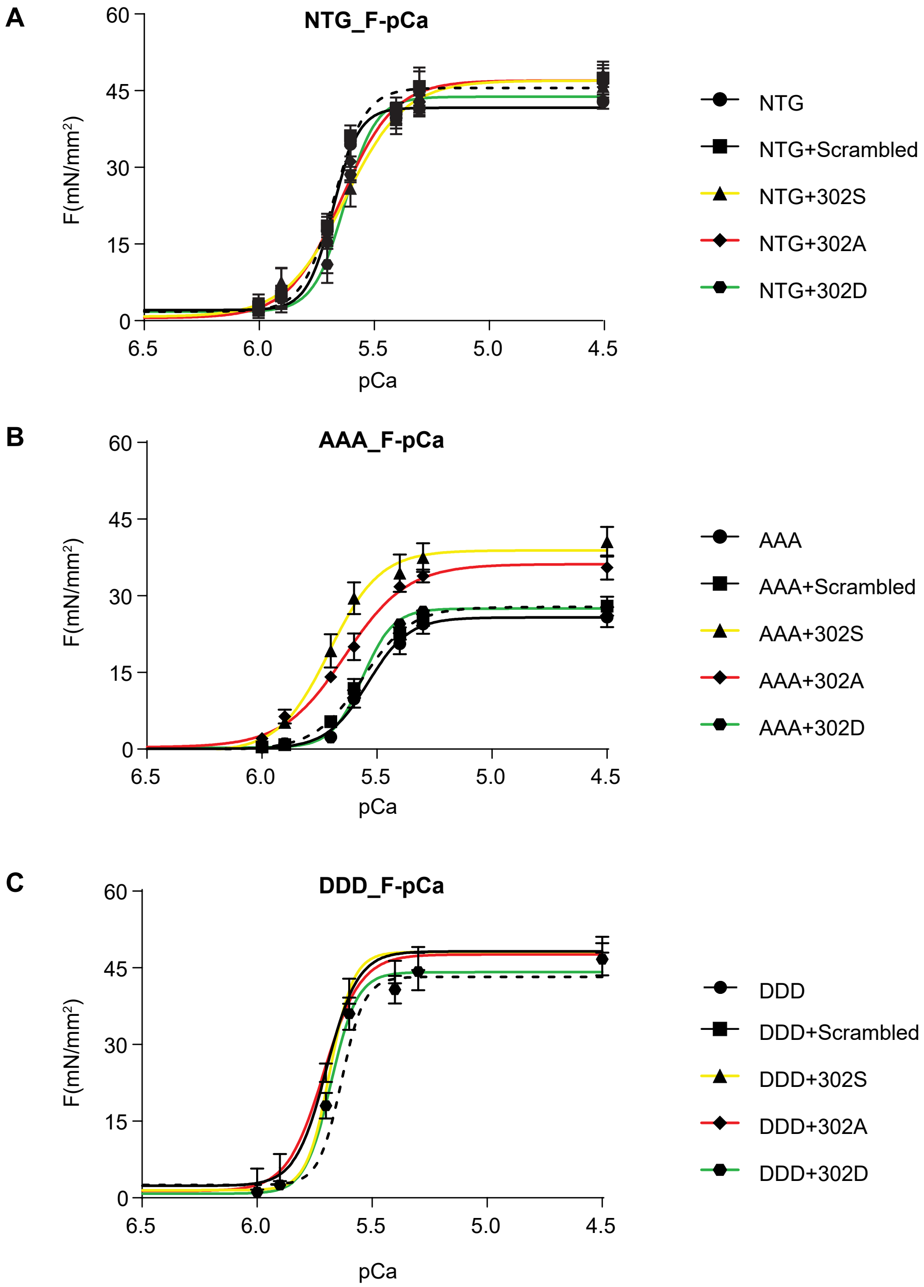

Supplementary Fig. S13. NTG, AAA and DDD muscle fiber force pCa curves in the presence of scrambled, 302S, 302A and 302D peptides. 302D and 302A peptides improved force generation in AAA muscle fibers (B) significantly, but did not change force geneartion in NTG and DDD muscle fibers (A & C).
